# A cellular mechanism contributing to pain-induced analgesia

**DOI:** 10.1097/j.pain.0000000000003315

**Published:** 2024-07-02

**Authors:** Federica Franciosa, Mario A. Acuña, Natalie E. Nevian, Thomas Nevian

**Affiliations:** Department of Physiology, University of Bern, Bern, Switzerland

**Keywords:** Inflammatory pain, Anterior cingulate cortex, Pyramidal neuron, Pain-induced analgesia, Descending modulation of pain, Periaqueductal gray, Excitability

## Abstract

The anterior cingulate cortex (ACC) plays a crucial role in the perception of pain. It is consistently activated by noxious stimuli and its hyperactivity in chronic pain indicates plasticity in the local neuronal network. However, the way persistent pain effects and modifies different neuronal cell types in the ACC and how this contributes to sensory sensitization is not completely understood. This study confirms the existence of 2 primary subtypes of pyramidal neurons in layer 5 of the rostral, agranular ACC, which we could classify as intratelencephalic (IT) and cortico-subcortical (SC) projecting neurons, similar to other cortical brain areas. Through retrograde labeling, whole-cell patch-clamp recording, and morphological analysis, we thoroughly characterized their different electrophysiological and morphological properties. When examining the effects of peripheral inflammatory pain on these neuronal subtypes, we observed time-dependent plastic changes in excitability. During the acute phase, both subtypes exhibited reduced excitability, which normalized to pre-inflammatory levels after day 7. Daily conditioning with nociceptive stimuli during this period induced an increase in excitability specifically in SC neurons, which was correlated with a decrease in mechanical sensitization. Subsequent inhibition of the activity of SC neurons projecting to the periaqueductal gray with in vivo chemogenetics, resulted in reinstatement of the hypersensitivity. Accordingly, it was sufficient to enhance the excitability of these neurons chemogenetically in the inflammatory pain condition to induce hypoalgesia. These findings suggest a cell type–specific effect on the descending control of nociception and a cellular mechanism for pain-induced analgesia. Furthermore, increased excitability in this neuronal population is hypoalgesic rather than hyperalgesic.

## Abstract 2

Pain-induced analgesia is mediated by increasing the excitability of a subpopulation of layer 5 pyramidal neurons in the anterior cingulate cortex projecting to the periaqueductal gray.

## 1. Introduction

The anterior cingulate cortex (ACC) is an important brain region for the emotional processing and cognitive control of pain.^10,14,79^ It shows increased activity during nociception and in chronic pain states.^1,2,40,67,77^ Interfering with signaling in this brain area can influence mechanical sensitivity as well as the affective component of pain.^29,54,61,71,74^ Inhibiting the ACC produces analgesic and anxiolytic effects,^23,43,46,64,84,86^ but counterintuitively increasing its activity can equally result in reduced pain behavior.^38,43,68^ These conflicting findings might be explained by the engagement of different neuronal subpopulations in the ACC with specific connectivity to other cortical and subcortical (SC) areas within the pain matrix.^26^ Of particular interest in this respect are layer 5 (L5) pyramidal neurons (PNs), which represent the main output neurons of the neocortex.^44^ These cells have been extensively studied at the functional and structural level predominantly in sensory cortices and in the medial prefrontal cortex,^35,53,75^ differentiating between 2 distinct subpopulations projecting to intratelencephalic (IT) or SC target structures.^13,33–35,37,41,72,83^ IT and SC neurons differ in their morphological and electrophysiological features, suggesting that they operate as distinct information channels serving different perceptual and behavioral functions. Particularly, cortical projections to the periaqueductal gray (PAG), a key region implicated in the descending modulation of pain^4,25,27,82^ and in placebo-induced analgesia^9,22,66^ by the rostral ventromedial medulla,^58^ might contribute not only to prolonged pain states but also to pain relief.^51,60^ Indeed, a loss in control of the descending pain modulatory pathway from the prelimbic cortex is involved in the development of chronic neuropathic pain.^19^ Furthermore, “heterotopic noxious conditioning stimulation” or “conditioned pain modulation,” phenomena based on the interference of a tonic painful stimulus with another phasic one, so that the latter is perceived as less aversive, is mediated by the descending pain modulatory system originating in the ACC.^5,70^

A detailed analysis of the L5 subtypes in the ACC has been lacking so far, as has an investigation into their contribution to the sensitization in peripheral inflammatory pain. It remains elusive whether persistent pain leads to uniform alterations in all PNs or if these changes are subtype specific. Hence, we tested the hypothesis that distinct neuronal subtypes in the ACC may contribute distinctly to the development of persistent inflammatory pain. We found differential and time-dependent plasticity in a subtype-specific manner. Intriguingly, repeated noxious stimulation, serving as a “counterirritation,” resulted in hypoalgesia and concomitantly increased excitability specifically in SC neurons. Accordingly, decreasing activity in the ACC-PAG pathway in this condition resulted in the reinstatement of the pain phenotype, whereas activating this pathway exhibited antinociceptive effects. Manipulating IT neurons had no impact, confirming a subtype-specific role in pain modulation. Thus, we discovered a novel mechanism for pain-induced analgesia, which is based on cellular changes in excitability in SC neurons.

## 2. Materials and methods

### 2.1. Animals

All experiments were approved by the veterinary office of the canton of Bern, Switzerland. Adult (8-15 weeks old), male, C57BL/6 mice were used for the experiments.

### 2.2. Complete Freund adjuvant model

Complete Freund's Adjuvant (CFA) (F5881; Sigma Aldrich; St. Louis, MO) (CFA 20 µL, 2% in 1:1 saline) was injected into the left hind paw of mice. Behavioral tests were performed before and 1 and 7 days after CFA injection to assess the inflammation-induced pain levels. The control group received an injection of saline solution.

### 2.3. Behavioural testing

Mice were placed individually on an elevated platform with a mesh floor and habituated for at least 30 minutes, unless otherwise specified. Mechanical sensitization was evaluated using the electronic von Frey test and the pinprick test. The plantar side of the hind paw was stimulated with calibrated von Frey filaments (IITC Life Science anesthesiometer; Woodland Hills, CA). For a single session, the mean withdrawal threshold of each paw was averaged from 6 repeated measurements. For the pinprick test, the plantar side of the hind paw was gently touched using a blunt 20-gauge attached to a filament, without penetrating the skin, and the responses were noted for 6 trials, with a 5-minute interval between the trials. The responses were scored as: 0 = paw lift; 1 = sustained lifting of the paw towards the body; 2 = strong lateral lifting above the level of the body; and 3 = flinching or licking of the affected paw (allodynia score).^20^ To investigate an associative learning effect for the pinprick conditioning, we performed von Frey testing at baseline, 1 and 7 days after saline/CFA injection in the same test context (room, mesh floor platform, and transparent plexiglass chambers). The daily pinprick stimulation was conducted either in the same context or in a different context in another room, where mice were placed on a different platform with a mesh floor and in a red plexiglass chamber.

To measure the extent of thermal sensitization, mice were placed on a hotplate (BIO-T2CT; Bioseb; Vitrolles, France) preset at 52°C, and animals were videotaped. The latency to show an affective response (flicking or licking the hind paw) was measured. A cutoff of 30 seconds was defined to prevent tissue damage. To study generalized thermal hypersensitivity, the heat-evoked tail withdrawal reflex was monitored. Two days before the first test session, mice were habituated to handling (light restraint in a soft cloth) and the tip of the tail (5 cm from distal) was immersed into room temperature water. On the test day, mice were lightly restrained, and the tail was submerged into 52°C water. Latency to response (flicking the tail out of the water) was measured using a stopwatch. Three tail withdrawal measurements were taken 10 minutes apart and averaged for a single data point for each animal. For chemogenetic experiments, saline or clozapine-*N*-oxide (CNO) (Cat No. 6329; Tocris; Zug, Switzerland) was injected i.p. before von Frey testing. Mice were placed in a chamber on a grid for 30 minutes habituation and subsequent von Frey testing 24 hours after CFA injection. Three hours later, saline i.p. as a control was administered before habituation and subsequent von Frey testing. After testing, mice were put back in their home cages. At day 7, animals were habituated, tested using von Frey, and put back in their home cages. Three hours later, CNO (10 mg/mL) was injected i.p. immediately before habituation, and testing was performed 30 minutes later.

### 2.4. Acute brain slice preparation

At different timepoints, mice were anesthetized with isoflurane and decapitated, and the brain was quickly removed and transferred to ice-cold oxygenated solution containing, in mM: 65 NaCl, 2.5 KCl, 1.25 NaH_2_PO_4_, 25 NaHCO_3_, 7 MgCl_2_, 0.5 CaCl_2_, 25 glucose, and 105 sucrose. Coronal slices of 300 µm thickness were cut from the tissue block with a vibratome (Microm/Thermo Scientific; Waltham, MA) and kept in artificial cerebrospinal fluid (aCSF) containing, in mM: 125 NaCl, 2.5 KCl, 1.25 NaH_2_PO_4_, 25 NaHCO_3_,1 MgCl_2_, 2 CaCl_2_, and 25 glucose, in a recovery chamber at 30°C for 30 minutes. Brain slices were stored at room temperature and oxygenated until further use. All brain slices had been tested behaviorally before.

### 2.5. Patch-clamp electrophysiology

After a 30-minute incubation at 30°C, slices were allowed to reach room temperature and recover for at least 30 minutes before being transferred to the recording chamber, superfused with 30 to 32°C aCSF. Whole-cell patch-clamp recordings were performed from L5 PNs in the rostroventral ACC (1.1-1.4 mm below the pial surface, 1.1-0.2 mm rostral to Bregma) on the contralateral side of the injured paw. Cells were identified using infrared gradient contrast video microscopy with a 40x objective (Leica Microsystems; Mannheim, Germany).^55^ Patch pipettes (5-9 MΩ) were pulled from thick-wall borosilicate capillaries (Hilgenberg; Malsfeld, Germany) and filled with intracellular solution containing, in mM: 130 potassium gluconate, 5 KCl, 10 HEPES, 10 sodium phosphocreatine, 4 Mg-ATP, 0.3 Na-GTP, and 0.2% biocytin. Recordings were performed using Dagan BVC-700A amplifiers (Dagan; Minneapolis, MN), and data were acquired with an ITC-16 board (Instrutech/HEKA; Reutlingen, Germany) and using Igor software (Wavemetrics; Lake Oswego, OR). Neurons were visualized with an IR CCD camera mounted on a Leica DM LFSA microscope equipped with light-emitting diodes (LEDs) for fluorescence excitation and infrared sample illumination (Thorlabs; Newton, NJ). Recordings were filtered at 5 kHz and digitized at 10 kHz. For the ex vivo validation of chemogenetic experiments, 10 µM CNO was added to the aCSF and bath-applied for 10 minutes before assessing the effects.

### 2.6. Morphology

At the end of ex vivo patch clamp experiments, the recording pipette was gently retracted, and the brain slice was fixed in 4% paraformaldehyde (Cat No. 1004960700; Sigma Aldrich) at 4°C overnight for reconstruction.^21^ After washing the slices with phosphate buffered saline (PBS) (3×, 10 minutes), they were permeabilized in PBS containing 2% Triton X-100 for 1 hour. Then slices were incubated with streptavidin-conjugated Alexa 488 (1:200) (Cat No. S11223; Thermo Fisher; Waltham, MA) or Alexa 405 (1:200) (Cat No. S32351; Thermo Fischer) in PBS containing 1% Triton X-100 (Cat No. X100-100ML; Sigma Aldrich). After washing with PBS (3×, 10 minutes), the processed slices were embedded in custom-made antifade based on Mowiol 4-88 (Cat No. 81381-50G; Sigma Aldrich) on microscopy slides. For morphological reconstruction, fluorescently labeled cells were imaged using a confocal microscope (SP8; Leica Microsystems) equipped with a white-light laser and 2 GaAsP detectors (HyD). Imaging was performed with a 20x objective (HC PL APO, 20×, NA 0.75 IMM CORR CS2; Leica Microsystems). All cells included in the analysis were checked for optimal filling in both apical and basal dendrites. Before tracing, cells were visually inspected and discarded if proximal branches of the dendrites appeared cut. Morphological parameters were quantitatively analyzed with Fiji software (ImageJ; NIH; Bethesda, MD), including somatic area, cross-section of apical dendrite measured at reported distance, the maximal horizontal and vertical field span of apical and basal dendrites (Max h, Max v), the number of basal dendrites (Den), the angle of the main bifurcation, the maximal branching order (Max order) for apical dendrites, and the number of oblique dendrites (Oblique). For each neuron, manual tracing was performed by following the dendrites from the soma to the pia. The mean arborization expansion was calculated as the area covered by the maximum horizontal extension and the maximum vertical expansion. The complexity of arborization was calculated by the Euclidean norm of the values given the horizontal and vertical maximal expansion for both apical and basal dendrites as well as the Max order, Den, and Oblique.

### 2.7. Electrophysiological analysis

Electrophysiological data were analyzed using a custom-written procedure in MATLAB (MathWorks; Natick, MA). To determine the input resistance, the cells were hyperpolarized by injection of a −300-pA current pulse of 600-millisecond duration. The steady-state voltage deflection as a function of the current applied was then evaluated. Membrane decay time constant tau was estimated fitting a mono-exponential function to the voltage response given the same current step. The sag ratio was calculated by:

$$\text{Sag}\;\text{ratio}=\frac{V_{\text{baseline}}-V_{\text{min}}}{V_{\text{baseline}}-V_{\text{steady‐state}}}$$

where V_baseline_ is the resting membrane potential (RMP), V_min_ is the minimum voltage reached after the hyperpolarizing current pulse, and V_steady-state_ is the median voltage recorded during the last 50 ms before the end of the stimulus. Voltage sag amplitude, in response to hyperpolarizing current pulses, was determined as the voltage difference between the peak hyperpolarization and the steady-state membrane potential. The rebound depolarization (RD) amplitude is the difference between the average resting membrane potential and the peak after −300 pA hyperpolarization in a 5 ms time window. To find the action potential (AP) threshold and the firing frequency, cells were depolarized by injection of 100 pA increasing current steps for 600 ms. The anteroposterior (AP) threshold was calculated by fitting a spline function to the values of the number of APs obtained given the current pulses. The value of current needed to evoke one AP (ie, the rheobase) was obtained. The firing frequency was calculated as the number of APs at each current step. Single AP features, such as AP threshold, amplitude, half-width, and the upstroke–downstroke ratio, were calculated using the first AP at rheobase. AP amplitude was defined as the peak relative to RMP. AP half-width was measured as the width at half-maximal amplitude. AP-firing adaptation was obtained when at least 10 APs were elicited in a single current pulse. Adaptation index was calculated as a ratio between the first interspike interval and the ninth (or the last) interval (ISI-9). AP bursts were identified in a 15-ms window at 200 pA of current injection. The number of AP in this window was used to determine the frequency of bursting.

### 2.8. Multidimensional morphological and electrophysiological analysis

We used various methods to analyse and classify the neuronal populations in the ACC. Initially, we used a tree classifier to determine the important variables that contribute to the separation between neuronal types. The classifier was trained using the features from the retrograde labelled dataset, specifically the morphological and electrophysiological variables. The importance of each variable was assessed using the “fittree” function in MATLAB, and the resulting importance values were sorted in ascending order. Nonzero importance values were then normalized, and the predictor names were sorted accordingly. To identify the significant predictors, we performed a *t* test for each predictor between 2 groups: IT and SC. The significant predictors were identified based on *P*-values < 0.05. Subsequently, we conducted a principal component analysis (PCA) on the significant predictors. The data were first standardized using *z*-scores, and then PCA was performed. We obtained 2 principal components, which captured the most significant variability in the data. To classify the neuronal types, we trained a logistic regression classifier, based on the PCA data. Cross-validation with 5 folds was performed to assess the performance of the classifier. The trained classifier was then used to predict the neuronal types in the validation set. The performance of the classifier was evaluated using the validation predictions and scores. The entire cross-validation process was repeated for each fold, and the predictions and scores were combined to obtain the final classification results.

These methods allowed us to characterize the neuronal populations in the ACC. The tree classifier, PCA, and logistic regression classifier provided valuable insights into the important variables, variability in the data, and classification of neuronal types, respectively. We then used the trained logistic regression model to predict the label of blindly recorded neurons. First, we grouped all recorded neurons and ran PCA using the significant features. Then we ran the classification model on the PCA data. In addition, the same principles were applied for data obtained from retrogradely neurons in CFA and saline conditions.

### 2.9. Virus injections

A standard stereotaxic frame (Kopf Instruments; Tujunga, CA) was used to take AP and mediolateral (ML) coordinates (relative to bregma) as well as dorsoventral (DV) coordinates (from the pial surface) for intracranial injections. Respectively, these coordinates were (in millimeter) +0.75, ±0.3, and −1.75 for ACC injections, AP: −4.3; ML: 0.5; DV: −2.2 for dorso-lateral PAG (dlPAG), and −4.3, +0.3, and −3.3 for the ventro-lateral PAG (vlPAG). Virus solutions containing AAV-retro2-hSyn1-chl-EGFP-T2a-iCre (Viral Vector Facility [VVF] of the University of Zürich, v146), AAV-retro2-hSyn-chl-mCherry-2A-iCre-WPRE-SV40p(A) (VVF, v147) (200 nL), AAV2-hSyn1-dlox-HA-hM4D(6F)-mCitrine (VVF, v93) (100 nL), AAV2-hSyn1-dlox-hM3D(Gq)-mCherry(rev)-dlox (VVF, v89) (100 nL) were slowly injected through heat-pulled glass pipettes connected to a Picospritzer pressure microinjector (100-200 nL/min; Parker Hannifin; Etoy, France). Viruses were injected unilaterally for electrophysiological and behavioral experiments. After surgery, viral constructs were allowed to express, and mice allowed to recover, for at least 4 weeks before experimentation.

## 3. Results

### 3.1. Two layer 5 pyramidal neuron subpopulations defined by different axonal projection patterns display distinct morphological and electrophysiological features

**Figure 1.**
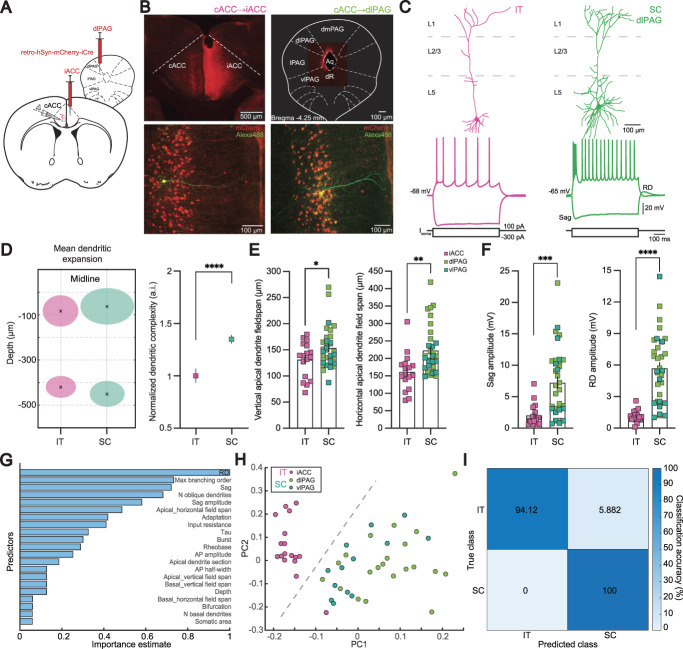
Identification of 2 types of pyramidal neurons in layer 5 of the ACC. (A) Sketch of strategy for targeted patch-clamp recordings from projection-specific ACC neurons. Retrogradely-labelling viral vectors were injected into the left iACC or the dlPAG to label IT and SC neurons, respectively, in the right contralateral ACC, where electrophysiological recordings were performed. (B) Confocal images of the injection sites in iACC (upper left) and dlPAG (upper right) and the corresponding recording sites in cACC of an IT (lower left) and SC neuron (lower right). Cells labelled with viral vectors appear in red and the recorded cells in green. (C) Representative pyramidal neuron reconstructions and example traces evoked by 600 ms current injections of −300 and 100 pA for an IT (magenta, left) and a SC neuron (green, right). (D) Mean arborization expansion (left) and complexity of arborization (right) of apical and basal dendrites in relation to the distance from midline for IT (magenta) and SC neurons (green). (E) Bar graph with individual values of vertical and horizontal field span of the apical dendrites. IT neurons are shown in magenta and SC neurons projecting to the dlPAG and vlPAG in light and dark green, respectively. (F) Bar graph with individual values of sag and rebound depolarization amplitudes. (G) Relative importance of significant prediction parameters based on the regression decision tree. (H) Principal component analysis performed on significant predictors from (G). (I) Confusion matrix computed from the logistic regression model validation from the PCA data. Statistical significance was determined by 2-tailed Student's *t* test. Error bars indicate SEM. *P < 0.05, **P < 0.01, ***P < 0.001, ****P < 0.0001. ACC, anterior cingulate cortex; dlPAG, dorso-lateral periaqueductal gray; PCA, principal component analysis; IT, intratelencephalic; SC, subcortical; vlPAG, ventro-lateral PAG.

Because the detailed composition of excitatory neuronal types in L5 of the ACC has not been documented so far, we first characterized differences in pyramidal cells (PNs) in this part of the brain. We focused on the rostroventral ACC, which is implicated in cortical pain processing.^76^ To label distinct L5 PN subpopulations and investigate their properties depending on their long-distance axonal projections, we injected a neuron-specific retrograde adeno-associated virus transducing mCherry (hSyn-retro-mCherry-iCre) into a cortical area, the right ACC, and into different regions of the PAG, a SC area. There, we targeted the dlPAG and vlPAG, respectively, as these 2 regions might be differentially involved in pain processing.^78^ In the left ACC, cell bodies expressing mCherry were found exclusively in L5 for all conditions (Figs. 1A and B, Fig. S1A, http://links.lww.com/PAIN/C80). To study the functional and structural diversity of these PNs, we used whole-cell patch-clamp recordings and subsequent morphological analysis of these neurons in naive mice.

Recordings were included in the analysis only if the resting membrane potential and input resistance were stable over time and if the pyramidal cell like morphology was intact. Quantitative analysis based on the morphometrics of 51 neurons revealed notable differences between the cortically and subcortically projecting subpopulations of PNs, with no distinction between SC neurons projecting to either the dlPAG or the vlPAG (Fig. 1C, Figs. S1 and S2, http://links.lww.com/PAIN/C80). The pooled SC population had distinct morphological features as compared with the IT neurons (Fig. 1, Fig. S2, and Table S1, http://links.lww.com/PAIN/C80). For example, SC neurons had larger pyramid-shaped somata than IT neurons and, on average, they had larger apical dendrites measured within approximately ∼10 µm distance from the soma as well as a significantly larger number of basal and oblique dendrites. The apical dendrites of SC neurons had a broader horizontal and vertical field span compared with the slender IT cells (Figs. 1D and E). Moreover, the branching patterns of apical, basal and oblique dendrites were more elaborate in SC neurons (Figs. 1C and D, Fig. S2C, http://links.lww.com/PAIN/C80), as illustrated by 2 representative, reconstructed neurons (Fig. 1C). Consequently, SC PNs showed a significantly higher level of complexity of both basal and apical dendrites (Fig. 1D). Notably, the distribution of IT and SC neurons within L5 of the ACC was similar (Fig. S2A, http://links.lww.com/PAIN/C80). The observed morphological differences between IT and SC neurons were in line with previous studies that have compared IT and SC cells in different cortical areas.^13,33–35,37,41,72,83^ These findings therefore suggest that different subtypes of PNs exist in the agranular ACC with distinct morphological features, similar to other cortical areas.

Next, we measured the intrinsic membrane properties to test whether the 2 populations also exhibited different electrophysiological characteristics. To perform whole-cell patch clamp, we prepared acute brain slices of the ACC from 8- to 10-week-old male mice previously injected with the retro tracer. Again, PNs projecting to the different parts of the PAG were indistinguishable in their electrophysiological features, suggesting homogenous properties for SC neurons in general (Fig. S1, http://links.lww.com/PAIN/C80). By comparison with the cortical projecting neurons, we found that a hyperpolarizing current injection of −300 pA and 600-ms duration evoked a prominent depolarizing voltage sag response in SC neurons because of the hyperpolarization-activated cation current (*I*_h_), whereas IT neurons had a significantly less pronounced sag (Fig. 1F, Fig. S2B, and Table S1, http://links.lww.com/PAIN/C80). In addition, SC neurons exhibited a significantly higher RD (Fig. 1F), with 32% of the neurons exceeding the action potential threshold. We found that both populations had a similar RMP (Fig. S2B, http://links.lww.com/PAIN/C80). Interestingly, despite their more complex morphology, the input resistance of SC and IT neurons was similar (Fig. S2B, http://links.lww.com/PAIN/C80), suggesting a similar absolute membrane conductance due to differential numbers of open ion channels in the resting state. Membrane capacitance and membrane time constant were also similar (Fig. S2B, http://links.lww.com/PAIN/C80). We found no significant difference in the rheobase current, that is the minimal current needed to elicit an action potential, measured in SC and IT neurons (Fig. S2B, http://links.lww.com/PAIN/C80). However, SC neurons exhibited a significantly higher firing rate over a broad range of current injections (Fig. S2B, http://links.lww.com/PAIN/C80). The majority of SC neurons (67%) (Fig. S2B, http://links.lww.com/PAIN/C80) were burst-spiking, as expected from previous descriptions of SC thick-tufted cells.^13,33,35^ Our results therefore indicate the presence of 2 distinct and nonoverlapping neuronal subpopulations in L5 of the ACC that possess different morphological and electrophysiological features depending on their projection target (Table S1, http://links.lww.com/PAIN/C80).

Subsequently, we used regression decision tree analysis to identify the key features that distinguished between SC and IT neurons. Our analysis revealed that the variables RD, max branching order, and sag ratio emerged as the most crucial factors in separating these neuronal types (Fig. 1G and Fig S3, http://links.lww.com/PAIN/C80). Among the initial pool of 28 predictors (Fig. S3, http://links.lww.com/PAIN/C80), we selectively retained only those exhibiting significant differences or a trend between the neuronal types, resulting in 21 predictors (Fig. 1G). To further validate the discriminatory power of these parameters, we trained a logistic regression model using the first 2 principal components (Fig. 1H). Our model demonstrated an accuracy of 95% during validation (Fig. 1I). As expected from the previous analysis, SC neurons projecting to the 2 regions of the PAG were classified as a homogenous group. These results underscore the high separability between the SC and IT neuronal populations, highlighting the potential of the identified morphological and electrophysiological features to serve as robust discriminators for the classification of any L5 PN.

### 3.2. Acute peripheral inflammation results in decreased activity of intratelencephalic and subcortical neurons

**Figure 2.**
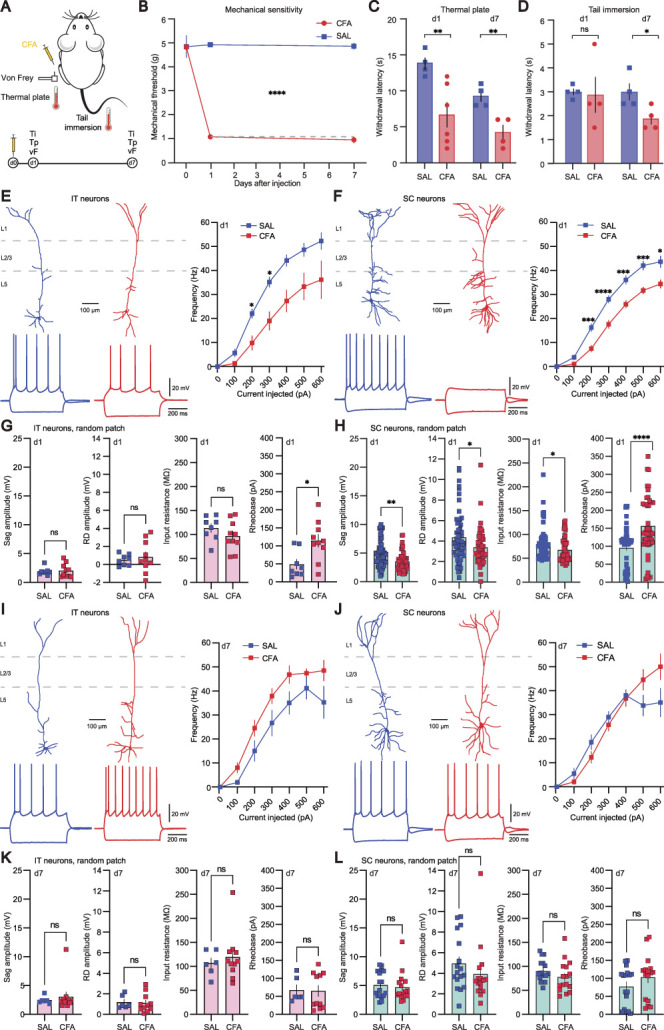
Acute peripheral inflammation results in decreased activity of IT and SC neurons. (A) Sketch and timeline of the CFA model of inflammatory pain used in the behavioural and electrophysiological experiments. (B) Mechanical threshold measured with an electronic von Frey device over time for CFA-injected (red) and saline-injected (SAL, blue) animals. (C) Thermal sensitivity test to heat (thermal plate at 52°C) for CFA-injected (red) and saline-injected (blue) animals. (D) Tail immersion test for CFA-injected (red) and saline-injected (blue) animals. Hypersensitivity to heat extended to the tail in CFA-treated mice at day 7. (E) Reconstructions of representative IT neurons from saline- and CFA-injected mice with their corresponding firing pattern evoked by a 600-ms current injection of −300 and 100 pA (left). Average F-I curves for the 2 conditions (right) at day 1. (F) Reconstructions of representative SC neurons from saline- and CFA-injected mice with their corresponding firing pattern evoked by a 600-ms current injection of −300 and 100 pA (left). Average F-I curves for the 2 conditions (right) at day 1. (G) Selected electrophysiological properties of IT neurons at day 1. (H) Selected electrophysiological properties of SC neurons at day 1. (I) Reconstructions of representative IT neurons from saline- and CFA-injected mice with their corresponding firing pattern evoked by a 600-ms current injection of −300 and 100 pA (left). Average F-I curves for the 2 conditions (right) at day 7. (J) Reconstructions of representative SC neurons from saline- and CFA-injected mice with their corresponding firing pattern evoked by a 600-ms current injection of −300 and 100 pA (left). Average F-I curves for the 2 conditions (right) at day 7. (K) Selected electrophysiological properties of IT neurons at day 7. (L) Selected electrophysiological properties of SC neurons at day 7. Statistical significance was determined by 2-way ANOVA (B), Mann–Whitney test (C and D), two-tailed Student *t* test (G, H, K, and L) and by a mixed model followed by Sidak multiple comparison test in F-I curves. Error bars indicate SEM. *P < 0.05, **P < 0.01, ***P < 0.001, and ****P < 0.0001. ANOVA, analysis of variance; IT, intratelencephalic; SC, subcortical.

After identifying 2 types of PNs in L5 of the ACC, we next examined their properties in the state of inflammation. We used the CFA model of inflammatory pain, where CFA was injected into the plantar pad of the hind paw of male mice. This procedure induces long-lasting arthritis-like pain, which shares several pathological features of human rheumatoid arthritis.^45,62^ We investigated the development of mechanical and thermal hyperalgesia at different timepoints of inflammatory pain (Figs. 2A–D). All CFA-injected mice developed strong mechanical and thermal hypersensitivity at the injected paw, as measured with the electronic von Frey (Fig. 2B) and the thermal plate test (Fig. 2C), respectively. Pain and distress of CFA mice, assessed with the tail immersion test, was detected only at day 7 (Fig. 2D).

To check whether peripheral inflammation influenced the excitability of PNs in L5 of the ACC, we performed ex vivo whole-cell patch-clamp recordings followed by morphological analysis of neurons from mice in the acute (d1) and in the sustained phase (d7) of inflammation. We blindly recorded 212 L5 neurons that were post hoc classified as IT or SC based on our trained logistic regression model (Fig. 1H and Fig. S4, http://links.lww.com/PAIN/C80). To validate that the classification method was also applicable in the inflammatory pain condition, we performed electrophysiological recordings and morphological analysis on projection-specific neurons from ACC to the contralateral ACC (IT neurons) and from the ACC to the vlPAG (SC neurons) 1 day after CFA injection (Figs. S4–S6, http://links.lww.com/PAIN/C80). We confirmed that our model had an accuracy above 90% in this case, suggesting that even if inflammatory pain altered some cellular parameters, the overall classification worked reliably. As to the projection-specific cells and the post hoc classified cells, we found that the morphological features of both neuronal subtypes were unaltered after 1 day of CFA injection (Figs. 2E and F, Figs. S5 and S7, http://links.lww.com/PAIN/C80). The electrophysiological parameters of IT neurons projecting to the contralateral ACC were unaltered after 1 day of inflammatory pain (Figs. S5 and S6B, http://links.lww.com/PAIN/C80), whereas the whole population of IT neurons showed a specific increase in the rheobase (Fig. 2G) and a corresponding right shift in the input current to AP frequency curve (F–I curves), with a significantly decreased firing rate at 200 and 300 pA current injected (Fig. 2E). All other electrophysiological parameters of the IT neuron population were unchanged in the acute inflammatory condition (Fig. 2G and Figs. S5–S7, http://links.lww.com/PAIN/C80). In contrast, SC neurons from CFA mice showed a significantly decreased sag and RD amplitude and a significantly higher rheobase, accompanied by a significantly lower input resistance than cells taken from sham animals (Fig. 2H). CFA injection also induced a right shift in the F-I curve with a significantly decreased firing rate over a wide range of current injections (Fig. 2F). These major changes in excitability were also present when looking at identified SC neurons projecting to the vlPAG (Fig. S5, http://links.lww.com/PAIN/C80). Altogether, these results show that in the acute phase of the inflammation, IT and SC PNs in L5 of the ACC display reduced excitability, but differential plasticity in other electrical properties (Figs. 2E–H).

Persistent pain beyond the acute phase seems to be correlated with the development of hyperexcitability of excitatory neurons in many brain areas of the pain-processing system. It has previously been shown that neuropathic pain induced by chronic constriction injury causes potentiation of intrinsic excitability in L5 PNs in the ACC^11^ and that inhibition of ACC activity leads to analgesic and anxiolytic effects in animal models of chronic pain.^23,43,46,64,84,86^ Therefore, we tested next whether the properties of PNs in L5 were altered in CFA-treated mice also at later stages of inflammation, 7 days after injury. We found that there were no major alterations in the morphology of both IT and SC neurons, but the latter class had a significantly larger number of basal dendrites as compared with saline control (Fig. S8, http://links.lww.com/PAIN/C80), which is consistent with other reports on morphological changes in persistent pain.^50,52^ In addition, both subtypes showed no difference in the passive membrane properties, input resistance, and rheobase between saline and CFA mice anymore as well as in the active membrane properties (Figs. 2I–L and Fig. S8, http://links.lww.com/PAIN/C80). Accordingly, similar firing rates were detected in neurons of both groups in response to 600 ms of depolarizing current injections (Figs. 2I and J). Overall, L5 neurons recorded from CFA mice at day 7 unexpectedly showed no significant changes in their biophysical properties, despite mice with inflammation still displaying increased mechanical and thermal sensitivity.

### 3.3. Repeated noxious stimulation of sustained peripheral inflammation increases excitability only in subcortical neurons

It has been widely observed in animal and human studies that 2 painful stimuli can interact with each other to reduce the perceived pain intensity for one of them.^48^ For example, a phasic noxious stimulus delivered to one part of the body is perceived as less painful in the presence of a tonic painful stimulus at a different site.^18^ This phenomenon is thought to depend on “diffuse noxious inhibitory control” involving the descending pain modulatory system, in particular the ACC, which exhibits increased activity in this case.^70^ We transposed this phasic, acute effect to a prolonged, repeated stimulation paradigm and hypothesized that this might induce long-lasting effects in pain sensitivity. Because of the observed transition from reduced to normal excitability in L5 PNs in inflammatory pain progression, we furthermore tested if such a repeated “counterirritation” would have an effect on the physiological parameters in the ACC.

**Figure 3.**
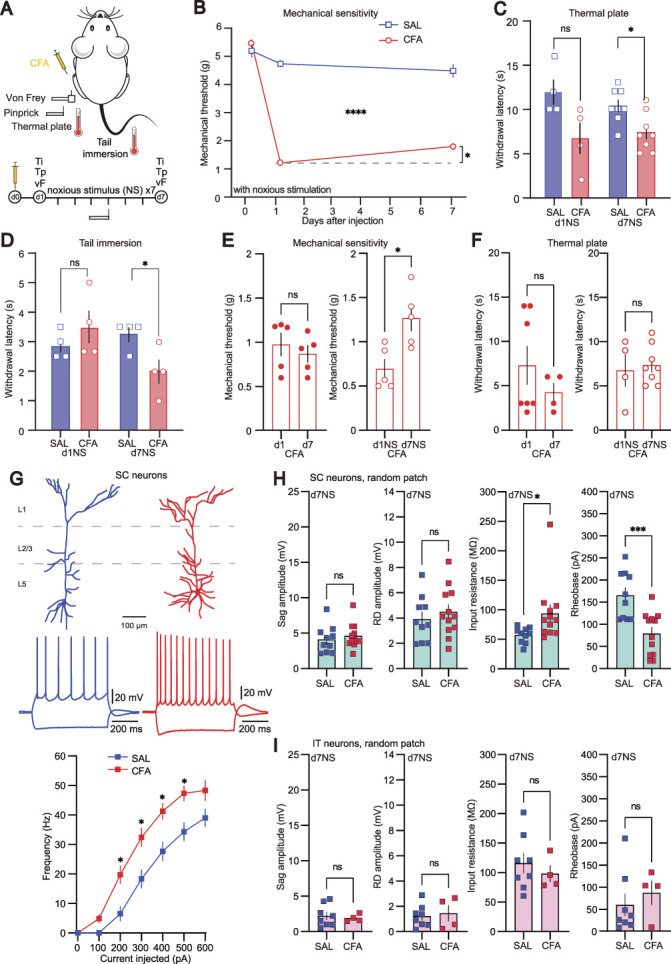
Repeated noxious stimulation of the hind paws during peripheral inflammation has analgesic effects and increases excitability specifically in SC neurons. (A) Sketch and timeline of the CFA model of inflammatory pain used in the behavioral and electrophysiological experiments. Animals were subjected to daily noxious pinprick stimulation to both paws in this set of experiments. (B) Mechanical threshold measured with an electronic von Frey device over time for CFA-injected (red) and saline-injected (SAL, blue) animals subjected to daily noxious stimulation by pinprick. Repeated noxious stimulation reduced the hypersensitivity in CFA-injected mice on day 7. Dashed line indicates withdrawal threshold at day 1. (C) Thermal sensitivity test to heat (thermal plate at 52°C) for CFA-injected (red) and saline-injected (blue) animals subjected to daily noxious stimulation by pinprick. Thermal sensitivity was not influenced in this condition. (D) Tail immersion test for CFA-injected (red) and saline-injected (blue) animals subjected to daily noxious stimulation by pinprick. Hypersensitivity of the tail to heat was not influenced in this condition. (E) Bar graphs of mechanical threshold of mice at d1 and d7 after CFA injection without intermediate interventions (left) and daily noxious pinprick stimulation for 1 week (right). (F) Bar graphs of thermal sensitivity of mice at d1 and d7 after CFA injection without intermediate interventions (left) and daily noxious pinprick stimulation for 1 week (right). (G) Reconstructions of representative SC neurons from saline- and CFA-injected mice subjected to daily pinprick with their corresponding firing pattern evoked by a 600-ms current injection of −300 and 100 pA (top). Average F-I curves for the 2 conditions at day 7 with daily pinprick stimulation (bottom). (H) Bar graphs of selected electrophysiological properties of SC neurons at d7 with daily pinprick stimulation. (I) Bar graphs of selected electrophysiological properties of IT neurons at d7 with daily pinprick stimulation. Statistical significance was determined by 2-way ANOVA followed by Sidak multiple comparison in (B and G), Mann–Whitney test (C and D), 2-tailed Student *t* test (E, F, H, and I). Error bars indicate SEM. *P < 0.05, **P < 0.01, ***P < 0.001, and ****P < 0.0001. ANOVA, analysis of variance; IT, intratelencephalic; SC, subcortical.

We injected mice with CFA or saline and gave a daily noxious stimulation to the hind paws using a pinprick needle (6×, 5 minutes interval) to both experimental groups for 7 days (Fig. 3A). As expected, CFA mice developed mechanical and thermal hyperalgesia of the injected hind paw as well as hypersensitivity to the tail rapidly after CFA injection (Figs. 3B–D). Pinprick stimulation was documented according to the allodynia score (Fig. S9, http://links.lww.com/PAIN/C80) that accounts also for coping mechanisms resulting from the stimulus.^20^ The repeated pinprick protocol in these animals significantly alleviated mechanical hypersensitivity as compared with the threshold measured at day 1 within the same group (Figs. 3B and E), whereas it did not have any impact on thermal sensitivity (Fig. 3F). These results suggest that the recurrent application of noxious stimulation counteracted the inflammation-induced hypersensitivity. As the testing of the mechanical withdrawal threshold was performed in the same context as the daily pinprick stimulation, it might have been possible that the reduction in hypersensitivity was a reflection of aversive conditioning that may lead to context-specific anti-nociception. To test this hypothesis, we performed von Frey testing in one context (context A), whereas noxious pinprick stimulation was performed daily in a visually different enclosure placed in a different room (context B; Fig. S10A, http://links.lww.com/PAIN/C80). This procedure should therefore prevent the association of sensory testing with the noxious experience. In this experimental condition, we still observed the significant increase in mechanical withdrawal threshold on day 7 (Fig. S10, http://links.lww.com/PAIN/C80), suggesting that the counterirritation indeed changed the mechanical sensitivity and was not based on conditioned aversive learning.

Next, we performed ex vivo recordings from L5 PNs from these animals on day 7 after final testing. No significant changes in the morphology of both IT and SC neurons in CFA or sham animals that had undergone the repeated noxious pinprick stimulation were found (Fig. S11, http://links.lww.com/PAIN/C80). IT neurons did not show any changes in their passive membrane properties nor in AP threshold (Fig. 3I, Figs. S11 and S12, http://links.lww.com/PAIN/C80). In contrast, SC neurons recorded from CFA mice subjected to noxious pinprick stimulation showed a significantly higher input resistance compared with neurons recorded from saline mice. This was accompanied by a significant decrease in the AP threshold (Fig. 3H). Correspondingly, the F-I curve showed a leftward shift for CFA neurons with a significant increase in firing rate over a wide range of current injections as compared with saline (Fig. 3G). Other biophysical properties such as RMP and time constant were similar between the 2 groups (Fig. S11, http://links.lww.com/PAIN/C80). These results suggest that the additional noxious stimulation in CFA mice leads to a reduction in mechanical sensitivity. In parallel, only the population of SC L5 PNs exhibited an increase in excitability. It is generally believed that increased neuronal excitability in the ACC is a hallmark of chronic pain. Yet, analgesic substances also lead to increased activity in this brain area.^1^ Thus, to evaluate the influence of the excitability of SC PNs on pain behavior and to establish a causal relation, we specifically manipulated their activity in the next set of experiments.

### 3.4. Subcortical neurons in the anterior cingulate cortex projecting to the periaqueductal gray modulate pain behaviour

**Figure 4.**
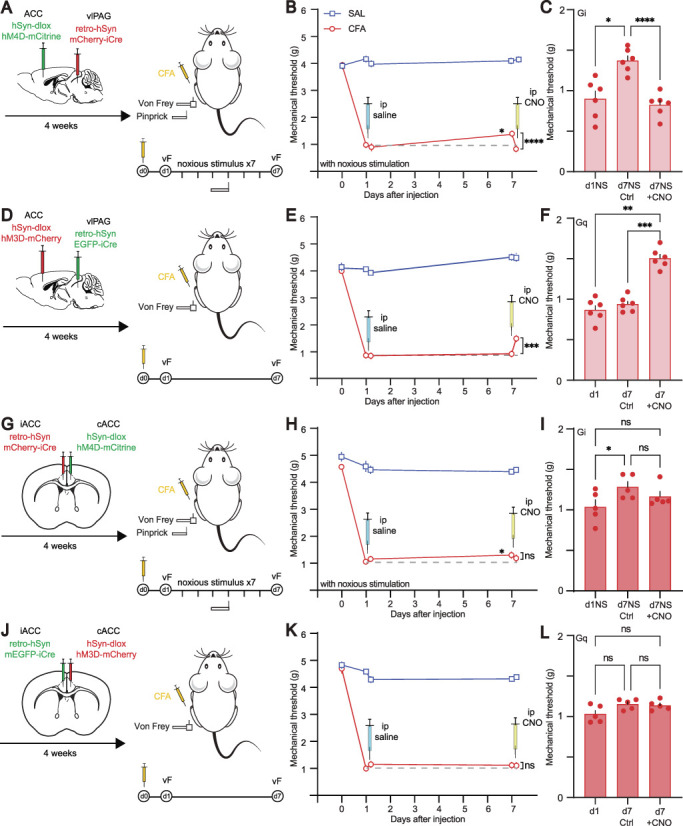
Anterior cingulate cortex-to-vlPAG projecting SC neurons modulate pain behavior. (A) Left: Viral targeting strategy to express hM4D-mCitrine in ACC neurons projecting to vlPAG for chemogenetic silencing of this SC pathway. Right: Sketch and timeline of the CFA model of inflammatory pain used for behavioral testing. Animals were subjected to daily noxious pinprick stimulation to both paws in this set of experiments. (B) Mechanical threshold measured with an electronic von Frey device over time for CFA-injected (red) and saline-injected (SAL, blue) animals subjected to daily noxious stimulation by pinprick. On day 7, mechanical sensitivity was assessed before and after i.p. injection of CNO to activate the inhibitory DREADD in the ACC-to-vlPAG pathway. Dashed line indicates withdrawal threshold at day 1. (C) Bar graph of mechanical threshold of mice at d1 and d7 after CFA injection subjected to daily pinprick stimulation and expressing the inhibitory DREADD before and after CNO injection. (D) Left: Viral targeting strategy to express hM3D-mCherry in ACC neurons projecting to vlPAG for chemogenetic activation of this SC pathway. Right: Sketch and timeline of the CFA model of inflammatory pain used for behavioral testing. Animals did not receive any intermediate interventions in this set of experiments. (E) Mechanical threshold measured with an electronic von Frey device over time for CFA-injected (red) and saline-injected (blue) animals subjected to no intermediate interventions. On day 7, mechanical sensitivity was assessed before and after i.p. injection of CNO to activate the excitatory DREADD in the ACC-to-vlPAG pathway. Dashed line indicates withdrawal threshold at day 1. (F) Bar graph of mechanical threshold of mice at d1 and d7 after CFA injection without intermediate interventions and expressing the excitatory DREADD before and after CNO injection. (G) Left: Viral targeting strategy to express hM4D-mCitrine in ACC neurons projecting to iACC for chemogenetic silencing of this IT pathway. Right: Sketch and timeline of the CFA model of inflammatory pain used for behavioral testing. Animals were subjected to daily noxious pinprick stimulation to both paws in this set of experiments. (H) Mechanical threshold measured with an electronic von Frey device over time for CFA-injected (red) and saline-injected (blue) animals subjected to daily noxious stimulation by pinprick. On day 7, mechanical sensitivity was assessed before and after i.p. injection of CNO to activate the inhibitory DREADD in the cACC-to-iACC pathway. Dashed line indicates withdrawal threshold at day 1. (I) Bar graph of mechanical threshold of mice at d1 and d7 after CFA injection subjected to daily pinprick stimulation and expressing the inhibitory DREADD before and after CNO injection. (J) Left: Viral targeting strategy to express hM4D-mCherry in ACC neurons projecting to iACC for chemogenetic activation of this IT pathway. Right: Sketch and timeline of the CFA model of inflammatory pain used for behavioral testing. Animals did not receive any intermediate interventions in this set of experiments. (K) Mechanical threshold measured with an electronic von Frey device over time for CFA-injected (red) and saline-injected (blue) animals subjected to no intermediate interventions. On day 7, mechanical sensitivity was assessed before and after i.p. injection of CNO to activate the excitatory DREADD in the cACC-to-iACC pathway. Dashed line indicates withdrawal threshold at day 1. (L) Bar graph of mechanical threshold of mice at d1 and d7 after CFA injection without intermediate interventions and expressing the excitatory DREADD before and after CNO injection. Statistical significance was determined by 2-way ANOVA followed by Sidak multiple comparison. Error bars indicate SEM. *P < 0.05, **P < 0.01, ***P < 0.001, and ****P < 0.0001. ACC, anterior cingulate cortex; IT, intratelencephalic; SC, subcortical; vlPAG, ventro-lateral periaqueductal gray.

We hypothesized that the altered excitability in the ACC by the pinprick stimulation affected the descending pain modulatory pathway, resulting in alterations in the pain response. The hyperexcitability observed only in the subcortically projecting neurons could account for their contribution to the “top-down” modulation of pain by the PAG. Using a chemogenetic approach, we manipulated the excitability of L5 neurons projecting to the PAG. We injected retro-Cre–dependent inhibitory and excitatory designer receptors exclusively activated by designer drugs (DREADDs) (hSyn-dlox-hM4D-mCitrine and hSyn-dlox-hM3D-mCherry, respectively) in the right ACC and a retro-Cre vector (retro-hSyn-mCherry-iCre and retro-hSyn-EGFP-iCre) in the right vlPAG, restricting the DREADD expression to the ACC neurons whose axons project to the vlPAG. Four weeks after the injection, inflammatory pain was induced in a group of mice (Figs. 4A and D). The animals expressing the inhibitory DREADDs (Figs. S14A–C, http://links.lww.com/PAIN/C80) were subjected to daily pinprick to induce the reduction of mechanical hypersensitivity. Seven days after CFA-induced inflammation, CNO was injected to inhibit the ACC-vlPAG neurons. We observed that mechanical hypersensitivity was significantly increased again to levels comparable with those measured at day 1 (Figs. 4B and C). Accordingly, we tested next if increasing excitability in this pathway in CFA-only treated animals transfected with an excitatory DREADD (Figs. S14D–F, http://links.lww.com/PAIN/C80) reduced mechanical sensitization. Indeed, activation of ACC-vlPAG connections by CNO injection led to a significant decrease in mechanical hypersensitivity (Figs. 4E and F). Performing the same manipulations in L5 neurons projecting to the dlPAG gave the same results. Reducing activity in these projection neurons after 7 days in CFA animals subjected to daily pinprick increased mechanical sensitivity, whereas enhancing activity in this pathway in CFA-only treated animals reduced mechanical sensitivity (Fig. S13, http://links.lww.com/PAIN/C80). Thus, manipulating ACC projections to either the vlPAG or the dlPAG had the same modulatory effect on mechanical sensitivity.

To assure specificity of SC neurons in influencing the pain threshold, we modulated the activity of IT neurons in a similar manner by expressing inhibitory or excitatory DREADDs in the contralateral ACC (Figs. 4G and J and Fig. S15, http://links.lww.com/PAIN/C80). We observed that the excitability level of IT neurons in L5 of the ACC was not relevant for the pain modulation at day 7. CFA-injected animals expressing inhibitory DREADDs subjected to pinprick showed no significant change in the mechanical threshold before and after CNO injection (Figs. 4H and I). Similarly, CFA-only treated mice expressing excitatory DREADDs also did not exhibit changes in their mechanical sensitivity by DREADD activation at day 7 (Figs. 4K and L). Thus, our results revealed a bidirectional modulation of mechanical sensitivity depending on the excitability state of L5 PNs in the ACC projecting to the PAG, suggesting that the state of neuronal excitability determines pain sensitivity. Counterintuitively, increased neuronal excitability was antinociceptive. This state could be induced either by repeated noxious conditioning or by activation of excitatory DREADDs in this specific pathway. Importantly, manipulating the activity of IT neurons had no effect on mechanical sensitivity, illustrating that specific pathways originating in the ACC are involved in analgesia but not the overall population excitability.

## 4. Discussion

Cortical processing depends on the interaction between distinct neuronal cell types in different cortical layers. Pyramidal cells in L5 are the principal output neurons of the neocortex and are thought to fall into 2 major categories defined by their axonal projections to SC vs IT targets.^39,57^ Accordingly, we also found these 2 classes of L5 neurons, suggesting that the ACC, even though it is an agranular cortex, obeys this universal rule as well. Some studies suggest a few further subclasses, which we could not find.^75^ The precise categorization depends on the anatomical and electrophysiological features being included into the analysis. SC neurons have been classified as large neurons with a more complex morphology than IT cells, referred to as thin or slender-tufted.^33,41^ SC neurons are intrinsically burst spiking with a large *I*_h_, whereas IT neurons are regular spiking with little *I*_h_, depending on a number of ionic conductances.^12,33,41,42,63^ In the granular primary somatosensory cortex, IT and SC neurons are mostly confined to layers 5A and 5B, respectively. We found that in the ACC, which is an agranular cortex, IT and SC neurons do not occupy specific layers but are intermingled. For the ACC, distinctions for L5 PNs have been determined mostly by their physiological properties, specifically the amount of *I*_h_ only.^49,65^ Other studies additionally included the projection targets.^47^ However, because of its variance, using a single parameter, like *I*_*h*_, might not be sufficient for an exact classification. This is relevant when studying pathological conditions ex vivo, in which the key parameter (eg, *I*_*h*_) undergoes plastic changes as is the case for chronic pain.^17,65^ We characterized L5 PNs in the ACC according to their projection specificity, morphology, and intrinsic physiological properties. This method of combining several cellular parameters allowed for classification with high accuracy even in the inflammatory pain condition and thus also in cases where the projection-identity could not be assured. The most important features determining their separability are the RD, the complexity of the apical dendrite, and the sag.

Chronic pain leads to a potentiation of intrinsic excitability in L5 PNs in the ACC,^11,65^ and a reduction of ACC activity is thought to produce analgesic, anxiolytic, and antidepressant effects.^23,24,43,64,84,86^ However, a distinction between different L5 subtypes had not been considered so far. The fact that callosal IT neurons integrate cortical information across hemispheres, and SC neurons exert top-down control over SC structures, let us hypothesize that IT and SC neurons might be differentially modulated during the development of persistent pain. Recently, it was shown that differences in synaptic modifications from the mediodorsal thalamus onto these 2 types of PNs and local interneurons inhibiting SC neurons are important for the aversive component of chronic pain.^47^ In addition, differential plasticity in PNs compared across cortical layers has been observed.^30,52,88^ These results, together with our findings, emphasize that specific subcircuits in the neocortex serve distinct functions in pain processing and undergo differential plastic changes in chronic pain.

We observed a decrease in neuronal excitability during the acute phase of inflammatory pain in both PN types, followed by a restoration to baseline levels after 1 week, while sensitization was maintained. The initial phase might reflect a homeostatic, adaptive response to the acute insult to counteract the heightened excitatory afferent drive.^73^ It could also reflect a physiological mechanism to transiently diminish the influence on the descending modulation of pain.^19^ Interestingly, IT neurons projecting to the ACC in the other hemisphere did not show any modifications in their cellular properties in the acute phase of inflammation, suggesting that differential plastic changes to pain can occur even within this subpopulation. In contrast, other studies have reported increased excitability in L5 PNs in the ACC in inflammatory pain, but this was measured at a much later timepoint,^69,86^ suggesting continued plasticity with disease progression. The ACC is a major hub for pain processing that is highly connected to other elements of the “pain matrix.” Thus, CFA-induced modulation of the main output neurons could have wide-ranging effects on other components of the matrix, thereby presumably altering the emotional/affective aspect of pain at a later stage, but not the nociceptive component that might be influenced by other pathways.^56^ In neuropathic pain, it is well established that PNs in the ACC become hyperexcitable^1,11,81,85,87^ and it is believed that these plastic changes contribute to the comorbid symptoms of anxiety and depression.^24,67,80^ Increased excitability is already present 1 week after nerve injury,^11^ which is in contrast to our observation here that at the same time point in inflammatory pain no apparent changes in excitability are evident. The increased excitability in the neuropathic pain condition could contribute to a masking of the hypersensitive state by tonic control of the descending pain modulatory system^19^ and highlights differences in plasticity depending on the etiology of the painful insult.

We found differential plasticity specifically in SC neurons, when applying repetitive noxious stimulation to both hind paws for several days in inflammatory pain. This treatment resulted in increased excitability in the SC neurons and a partial alleviation of the allodynic phenotype. Decreasing excitability of SC neurons projecting to the PAG in this condition with an inhibitory DREADD reinstated the mechanical sensitization. This demonstrated a causal relation between excitability levels in SC neurons and pain behavior, and it is consistent with a cortical control of descending modulation of pain relayed by the PAG. The activation of the vlPAG exerts inhibitory effects on spinal cord activity by innervating descending projections involving the rostral ventromedial medulla (RVM).^3,58^ In addition, the dlPAG has been suggested to be involved in analgesia,^36^ which fits to our observation that both vlPAG and dlPAG had the same effect on mechanical sensitivity. Accordingly, increasing excitability in the ACC-to-PAG pathway in animals suffering from inflammatory pain without prior treatment had analgesic effects. It was recently demonstrated that the prefrontal cortex can exert a similar control on nociception using the PAG,^19^ suggesting that multiple cortical brain areas of the pain matrix can be the origin of the descending modulation of pain. The increase in excitability in SC neurons can enhance the activity of the PAG that dampens the afferent nociceptive drive. The increased excitability of L5 PNs observed in other studies^69,86^ seems to be at odds with this hypothesis but might reflect that specific subsets of PNs in the ACC subserve different functions and that increased excitability and activity in the ACC might not be an indicator for the sensitization of the nociceptive system but possibly for other comorbid symptoms like anxiety or depression. Accordingly, we have recently demonstrated that the analgesic drug gabapentin results in increased activity in the ACC in vivo.^1^ Thus, our findings suggest that SC neurons in L5 of the ACC are part of a descending pain-modulatory circuit, and therefore, stimulation or inhibition of these neurons might be expected to suppress or enhance responses to painful stimuli in mouse models of persistent pain. The lack of effect on IT neurons suggests that not the overall excitability in the ACC but the plasticity of projection-specific neurons is the decisive factor in pain sensitization. It has been widely observed in animals as well as in humans that 2 painful stimuli can interact with each other to reduce the perceived pain intensity for one of them.^48^ Our results suggest that specific noxious stimulation patterns can induce a sustained analgesic effect. We propose a new cellular mechanism, which is based on the increase in excitability in SC neurons controlling the descending inhibition of pain by the PAG. This presumably long-lasting plasticity of intrinsic excitability for pain relief in our case is different from other reported pain-induced analgesic effects, like “diffuse noxious inhibitory control,”^6,7,70^ “noxious stimulus-induced analgesia,”^8,31^ or stress-induced analgesia,^16,28^ which are generally more transient, engage different pain pathways and cellular mechanisms.^15,32,59^ It should be noted that our results only apply to male mice and may not be generalizable to females as cellular mechanisms leading to inflammatory pain might be different.

## 5. Conclusion

Our study newly classifies 2 PN subtypes in the ACC and demonstrates that inflammatory pain induces distinct electrophysiological modifications in SC neurons compared with the IT subtype. Furthermore, our results provide novel insights into the mechanisms underlying pain-induced analgesia by revealing an intrinsic cellular plasticity mechanism that alleviates sensitization. These findings suggest that manipulating specifically the activity of SC neurons could have therapeutic implications for pain management. Particularly, physical therapies like mechanical, heat, and transcutaneous electrical nerve stimulation could be used to induce these long-lasting plastic changes. It needs to be evaluated which stimulation protocols and which modality might prove most effective. Overall, this study makes a valuable contribution to our understanding of the role of distinct subclasses of L5 PNs in the ACC and their significant differential involvement in pain processing.

## Conflict of interest statement

The authors have no conflicts of interest to declare.

## Supplementary Material

SUPPLEMENTARY MATERIAL

## Supplemental digital content

Supplemental digital content associated with this article can be found online at http://links.lww.com/PAIN/C80.
