# Supplementary material for: A cellular mechanism contributing to pain-induced analgesia
Source: Pain. 2024 Jul 2;165(11):2517–29. doi: 10.1097/j.pain.0000000000003315 (PMC11474934; doi:10.1097/j.pain.0000000000003315)
Supplement: SUPPLEMENTARY MATERIAL [file jop-165-2517-s002.pdf]

| Morphology | IT n=17 | SC n=34 | *p value* | |
|---|---|---|---|---|
| Depth in L5 (μm) | -418.8 ± 13 | -446.6 ± 8 | *p* = 0.07 | |
| Distance from soma to bifurcation (μm) | 268.1 ± 11 | 301 ± 14 | *p* =0.13 | |
| Somatic perimeter (μm) | 48.9 ± 1.9 | 55 ± 1.2 | *p* = 0.007 | ** |
| Somatic area (μm²) | 157.2 ± 11 | 198.2 ± 9 | *p* = 0.009 | ** |
| Apical dendrite section (μm) | 2.3 ± 0.1 | 2.7 ± 0.1 | *p* =0.008 | ** |
| Horizontal field span of basal dendrite (μm) | 143.2 ± 14 | 145.7 ± 6 | *p* = 0.8 | |
| Vertical field span of basal dendrite (μm) | 96.9 ± 8 | 109.8 ± 6 | *p* = 0.2 | |
| Number of basal dendrites | 4.8 ± 0.4 | 6.3 ± 0.2 | *p* = 0.001 | *** |
| Horizontal field span of apical dendrite (μm) | 161 ± 13 | 222.4 ± 12 | *p* = 0.002 | ** |
| Vertical field span of apical dendrite (μm) | 131 ± 8 | 153 ± 7 | *p* = 0.04 | * |
| Bifurcation angle (deg) | 36.5 ± 2.7 | 46.5 ± 2.5 | *p* = 0.02 | * |
| Maximal branching order | 3.5 ± 0.3 | 6.2 ± 0.3 | *p* <0.0001 | **** |
| Number of oblique dendrites | 5.7 ± 0.7 | 8.8 ± 0.5 | *p* = 0.001 | *** |
| **Electrophysiology** | | | | |
| Resting membrane potential (mV) | -66 ± 1.2 | -64 ± 0.7 | *p* = 0.1 | |
| Capacitance (pF) | 127.5 ± 49 | 122 ± 17 | *p* =0.9 | |
| Time constant (ms) | 14.2 ± 2 | 12.0 ± 1 | *p* = 0.3 | |
| Sag ratio | 1.09 ± 0.1 | 1.3 ± 0.02 | *p* <0.0001 | **** |
| Sag amplitude (mV) | 2 ± 0.4 | 7 ± 0.9 | *p* = 0.0004 | *** |
| Input resistance (MΩ) | 155 ± 19 | 124 ± 11 | = 0.14 | |
| Rebound depolarization amplitude (mV) | 1.1 ± 0.1 | 5.7 ± 0.5 | *p* <0.0001 | **** |
| Rheobase (pA) | 95 ± 24 | 73 ± 24 | *p* = 0.05 | |
| AP amplitude (mV) | 103 ± 2.5 | 96 ± 3 | *p* = 0.05 | |
| AP half-width (ms) | 1.3 ± 0.9 | 1.2 ± 0.04 | *p* = 0.08 | |
| Adaptation | 0.2 ± 0.04 | 0.4 ± 0.07 | *p* = 0.04 | * |
| Depolarization velocity (mV/ms) | 193 ± 16 | 222 ± 9 | *p* = 0.4 | |
| Hyperpolarization velocity (mV/ms) | 67.8 ± 5 | 75 ± 7 | *p* = 0.4 | |
| DAP amplitude (mV) | 3.6 ± 0.8 | 3.3 ± 0.6 | *p* = 0.8 | |
| Burst frequency (Hz) | 175 ± 11.7 | 173 ± 16 | *p* = 0.9 | |
| Percentage of neurons with rebound spiking | - | 32% | | |

**Table S1. Morphological and electrophysiological values of IT and SC PNs.** Values are mean ± SEM, with sample size indicated above the colums. *P*-values were determined by unpaired two-tailed Student's t-test. * p < 0.05, ** p < 0.01, *** p < 0.001, **** p < 0.0001.

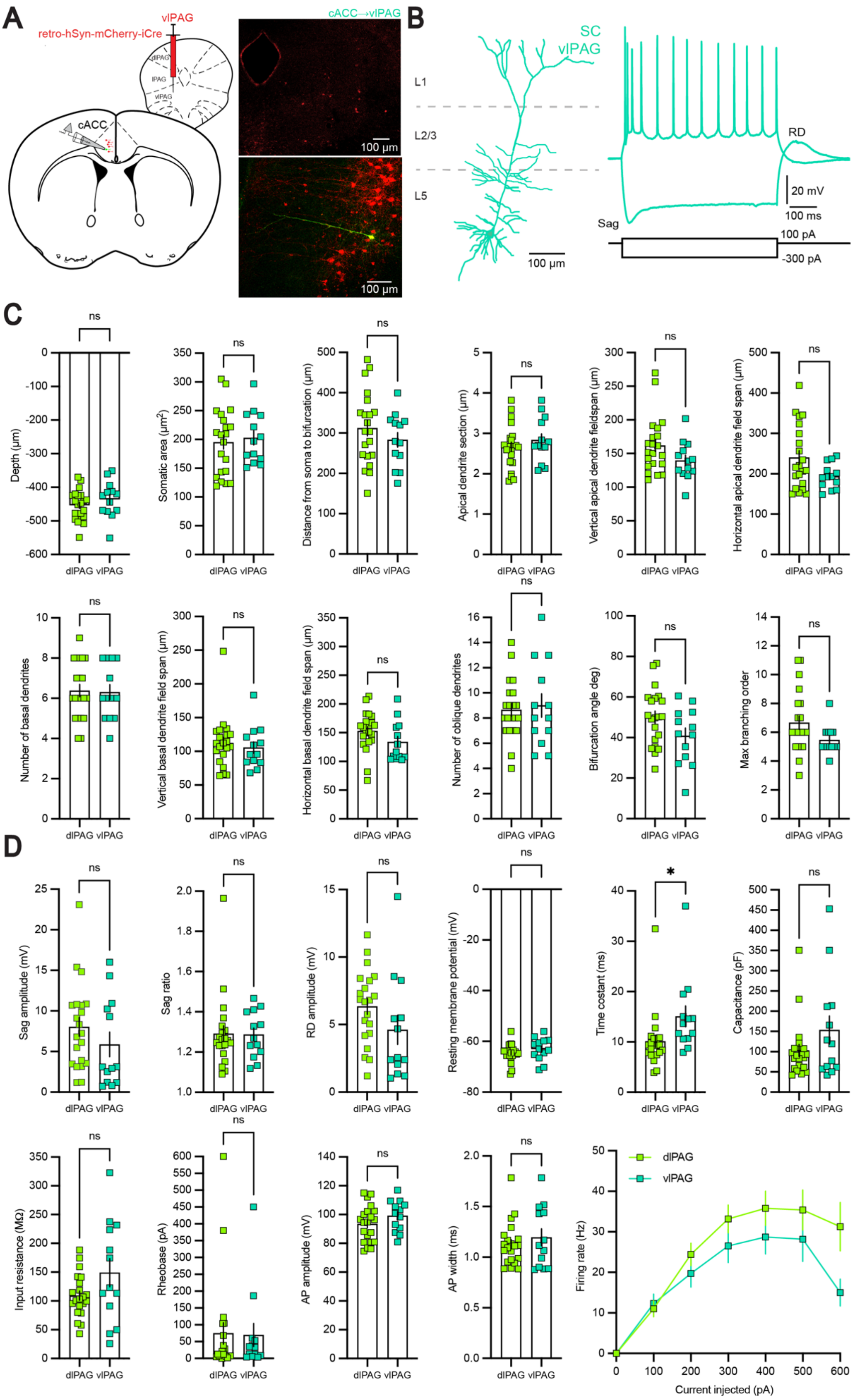

**Figure S1. Comparison of two identified subpopulation of SC neurons projecting to the dlPAG and vlPAG. (A)** Left, sketch of strategy for targeted patch-clamp recordings from projection-specific ACC neurons. Retrogradely-labelling viral vectors were injected into the vlPAG to label these specific SC neurons in the ACC, where electrophysiological recordings were performed. Right, confocal images of the injection site in vlPAG (upper right) and the corresponding recording sites in cACC of a SC neuron (lower right). Cells labelled with viral vectors appear in red and the recorded cell in green. **(B)** Representative pyramidal neuron reconstruction (left) and example traces (right) evoked by 600 ms current injections of -300 pA and 100 pA for a SC neuron projecting to vlPAG. **(C)** Bar graphs of morphological parameters comparing neurons from dlPAG (light green) and vlPAG (dark green). **(D)** Bar graphs of elctrophysiological parameters comparing neurons from dlPAG (light green) and vlPAG (dark green) Statistical significance was determined by two-tailed Student's t-tests. Error bars indicate SEM. * $p < 0.05$.

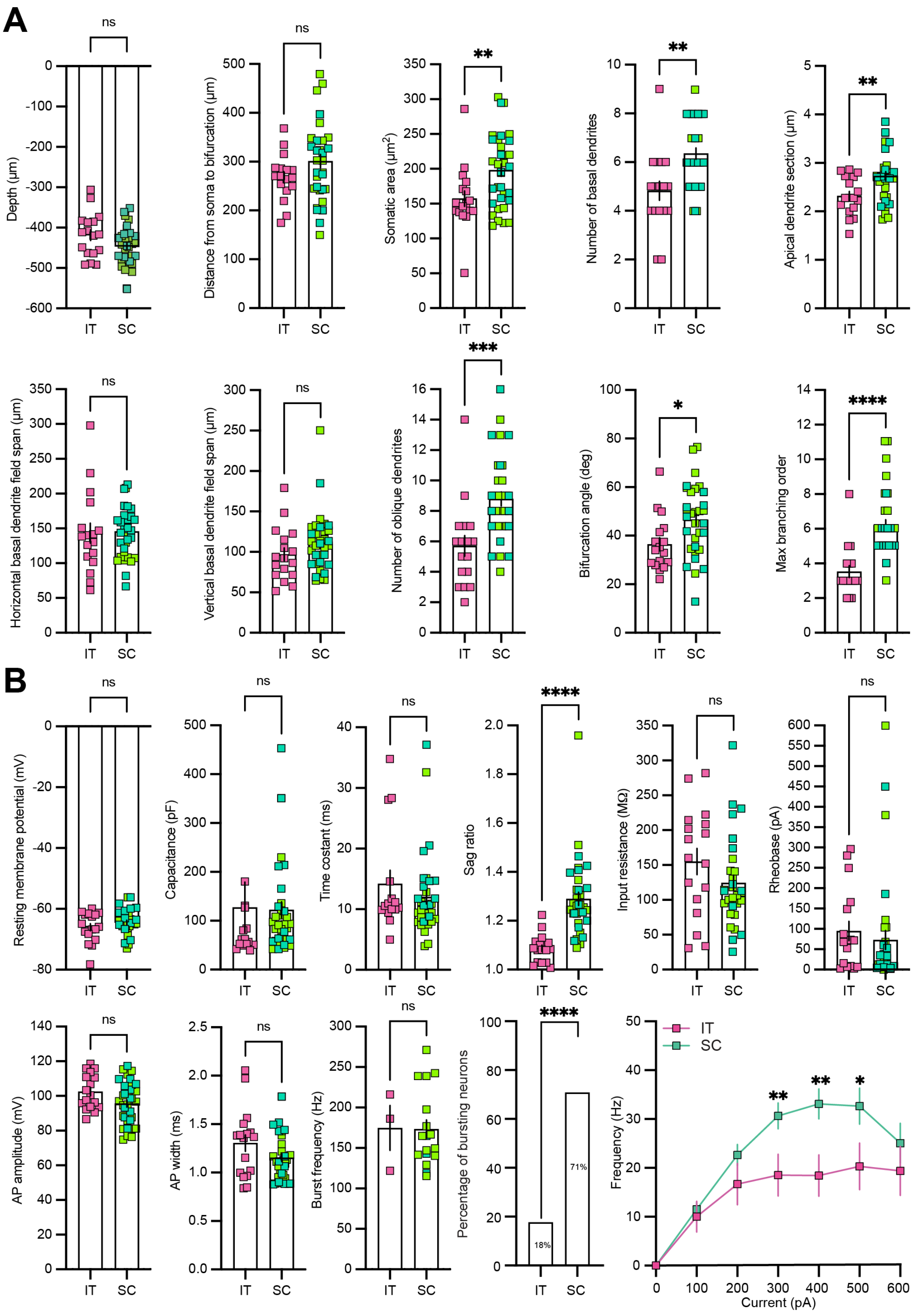


**Figure S2. Comparison of L5 IT and SC PNs. (A)** Bar graphs of morphological parameters comparing L5 PNs projecting to the ACC (IT, magenta) and subcortically (SC) to the dlPAG (light green) and vlPAG (dark green). **(B)** Bar graphs of electrophysiological parameters for IT and SC projecting PNs. Statistical significance was determined by two-tailed Student's t-test, by Fisher exact test for comparing percentage of bursting neurons and by a mixed-model followed by Bonferroni's *post hoc* test for the frequency-current relationship. Error bars indicate SEM. * $p < 0.05$, ** $p < 0.01$, *** $p < 0.001$, **** $p < 0.0001$.

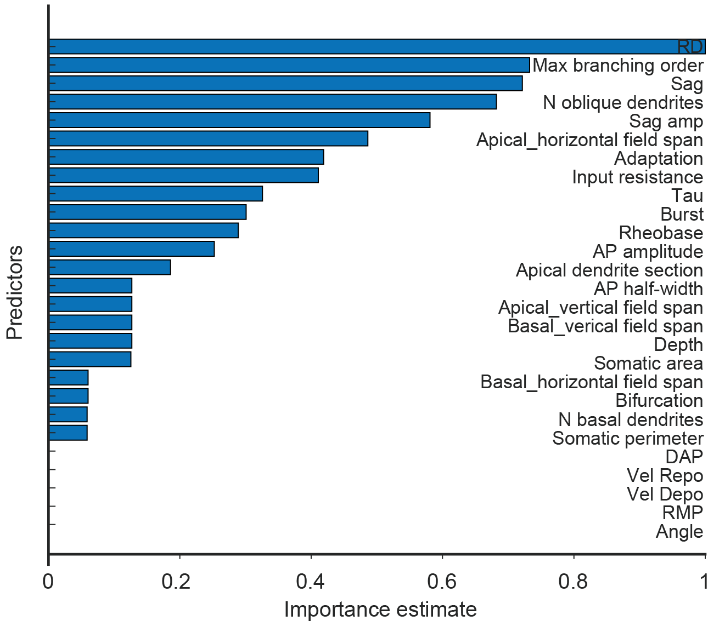


**Figure S3. Relative importance of all prediction parameters based on the regression decision tree.**

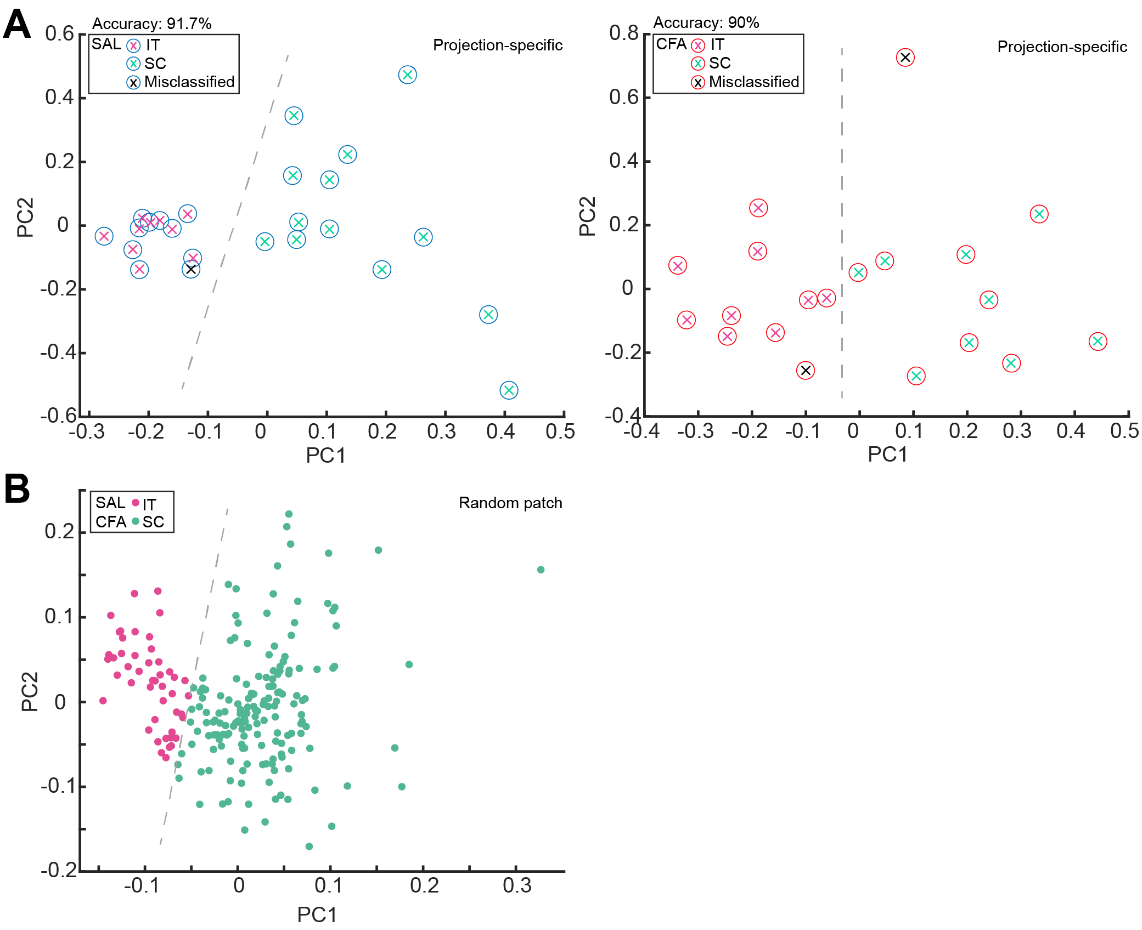


**Figure S4._Principal components analysis of L5 PNs with known projection targets one day after saline or CFA injection and application of this method for randomly patched neurons. (A)** Principal component (PC) analysis comprising the morphological and electrophysiological features of projection specific PNs from saline (blue, upper graph) and CFA (red, lower graph) treated animals based on the logistic regression model. IT and SC PNs are indicated by magenta and green crosses respectively. Black crosses indicate misclassified neurons. Dashed lines indicate the separation of the two classes, illustrating a high degree of accuracy using this method. **(B)** Principal component analysis for randomly patched neurons from saline and CFA treated animals classified as IT (magenta) and SC (green) PNs based on the logistic regression model.

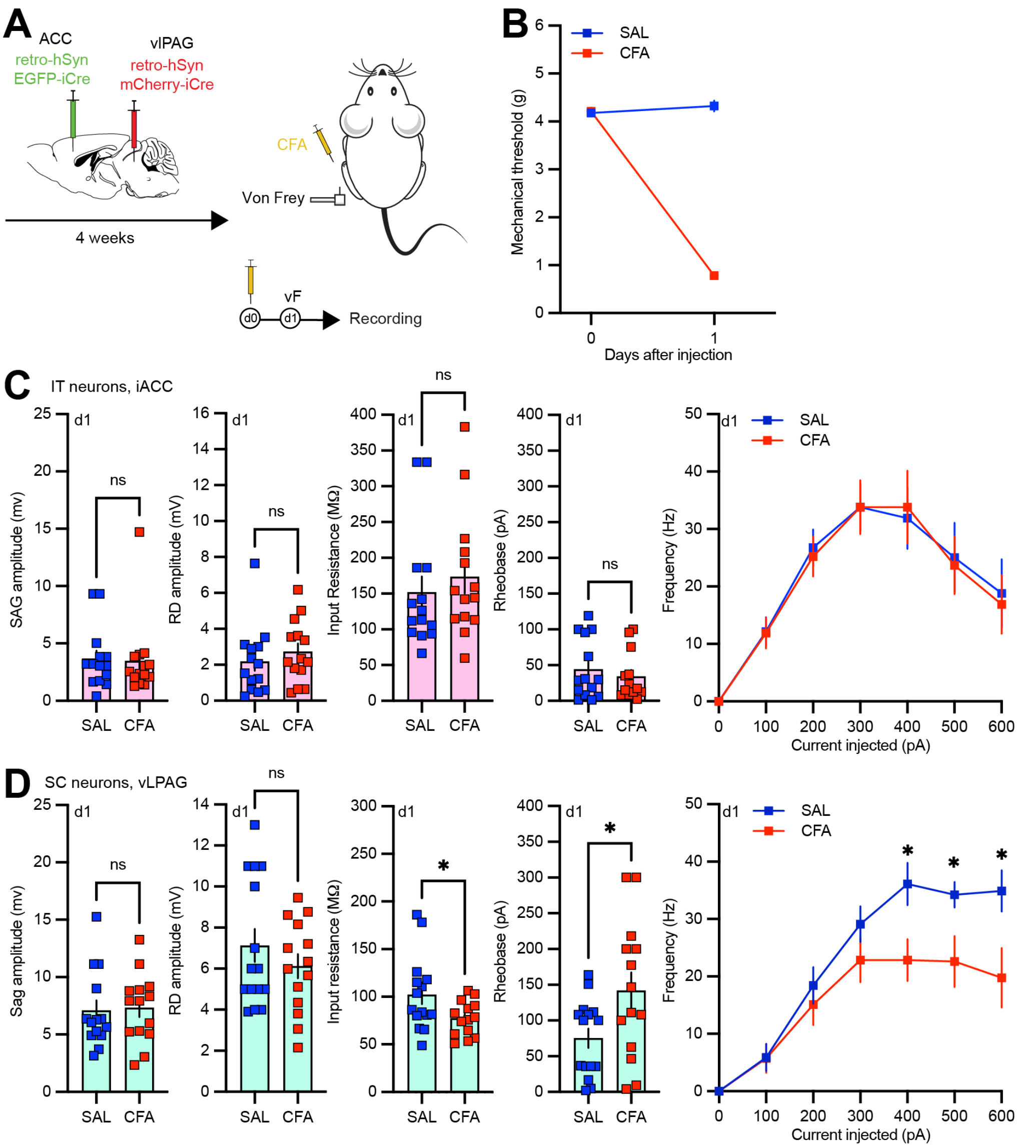


**Figure S5. Comparison of the most important electrophysiological features of identified IT and SC neurons one day after saline or CFA injection. (A)** Left, Sketch of strategy for targeted patch-clamp recordings from projection-specific ACC neurons. Right, sketch and timeline of the CFA model of inflammatory pain used in the behavioral and electrophysiological experiments. **(B)** Mechanical threshold measured with an electronic von Frey device over time for CFA (red) and saline (SAL, blue) injected animals. **(C)** Electrophysiological parameters of IT neurons for saline (blue) and CFA (red) injected animals. **(D)** Electrophysiological parameters of SC neurons for saline (blue) and CFA (red) injected animals. Statistical significance was determined by two-tailed Student's t-tests and by a mixed-model followed by Bonferroni's *post hoc* test for the frequency-current relationships. Error bars indicate SEM. * $p < 0.05$.

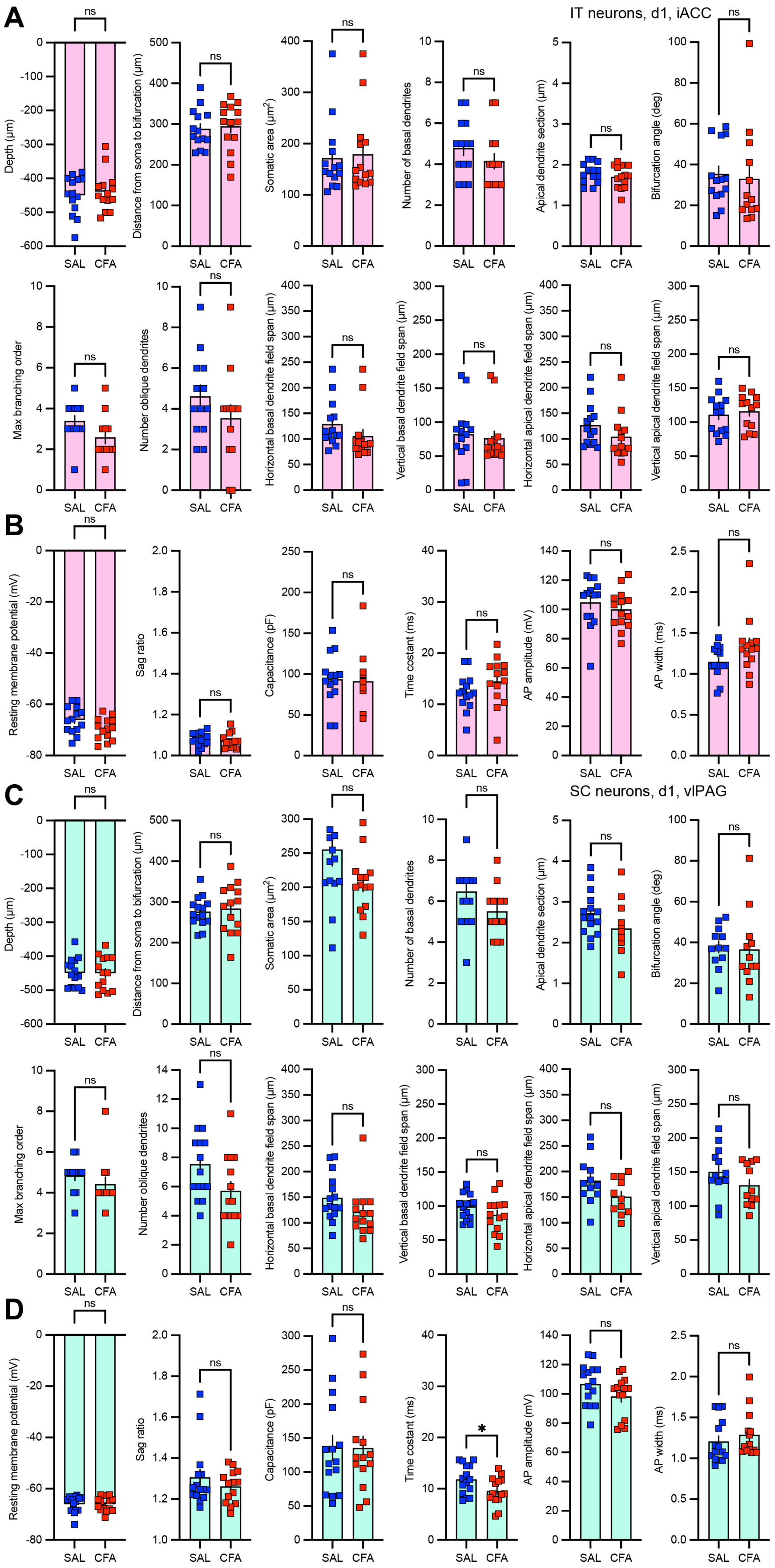


**Figure S6. Comparison of morphological and electrophysiological features of identified IT and SC neurons one day after saline or CFA injection. (A)** Bar graphs of morphological parameters of IT neurons for saline (blue) and CFA (red) injected animals. **(B)** Bar graphs of electrophysiological parameters of IT neurons for saline (blue) and CFA (red) injected animals. **(C)** Bar graphs of morphological parameters of SC neurons for saline (blue) and CFA (red) injected animals. **(D)** Bar graphs of electrophysiological parameters of SC neurons for saline (blue) and CFA (red) injected animals. Statistical significance was determined by two-tailed Student's t-tests. Error bars indicate SEM. * $p < 0.05$.

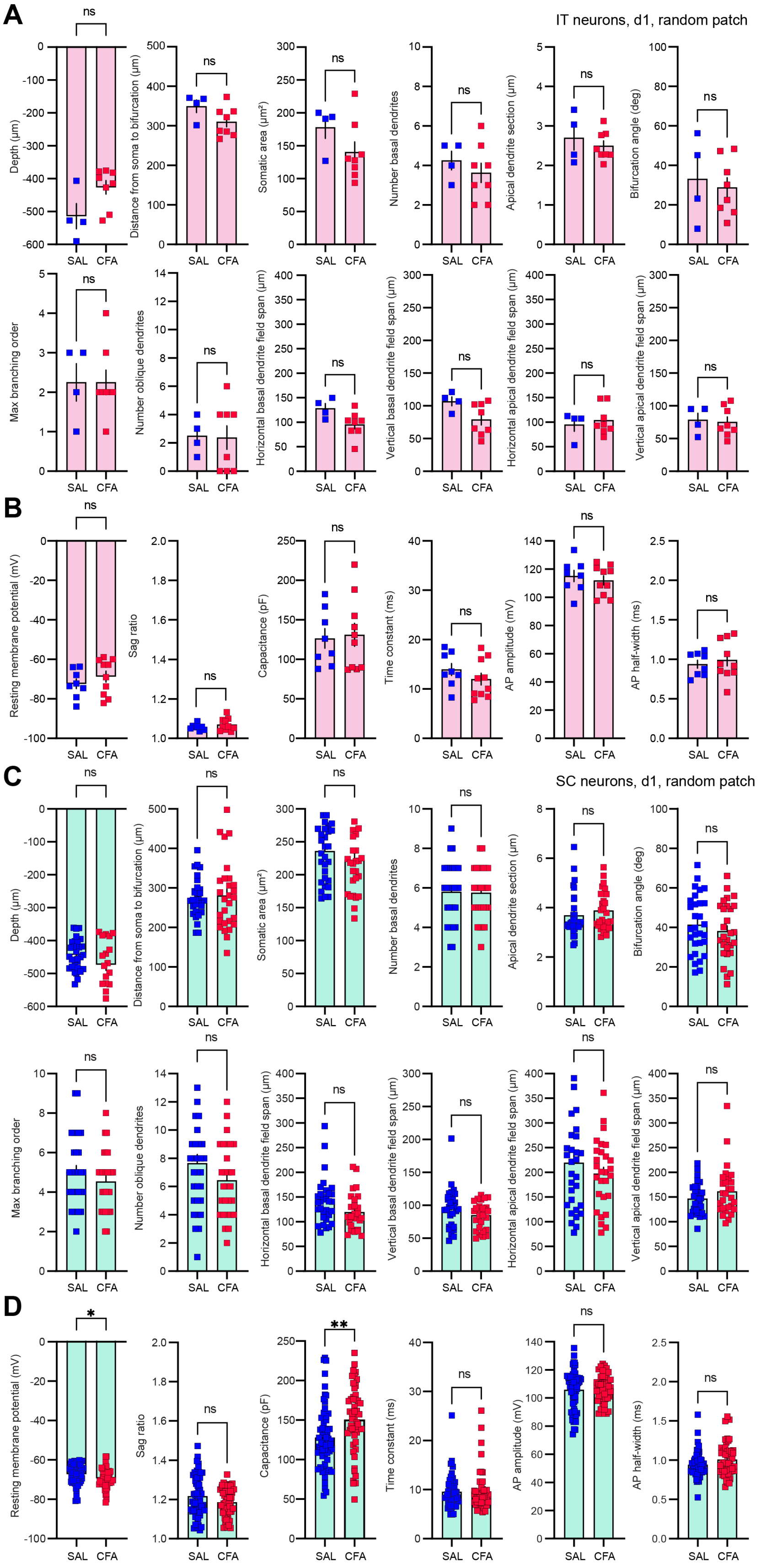


**Figure S7. Comparison of morphological and electrophysiological features of randomly patched PNs one day after saline or CFA injection that were post-hoc classified as IT and SC neurons. (A)** Bar graphs of morphological parameters of IT neurons for saline (blue) and CFA (red) injected animals. **(B)** Bar graphs of electrophysiological parameters of IT neurons for saline (blue) and CFA (red) injected animals. **(C)** Bar graphs of morphological parameters of SC neurons for saline (blue) and CFA (red) injected animals. **(D)** Bar graphs of electrophysiological parameters of SC neurons for saline (blue) and CFA (red) injected animals. Statistical significance was determined by two-tailed Student's t-tests. Error bars indicate SEM. * $p < 0.05$, ** $p < 0.01$.

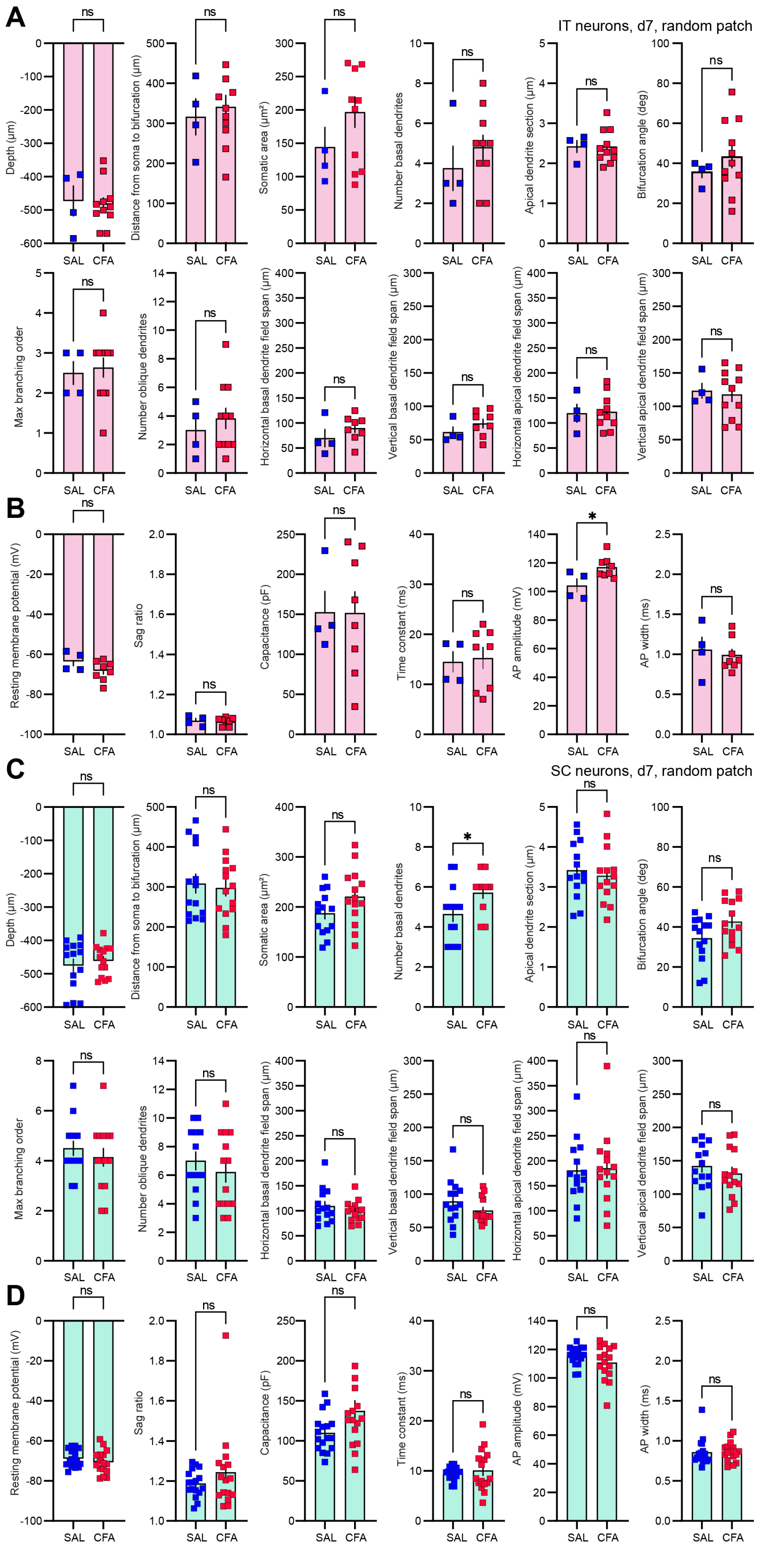


**Figure S8. Comparison of morphological and electrophysiological features of randomly patched PNs 7 days after saline or CFA injection that were post-hoc classified as IT and SC neurons. (A)** Bar graphs of morphological parameters of IT neurons for saline (blue) and CFA (red) injected animals. **(B)** Bar graphs of electrophysiological parameters of IT neurons for saline (blue) and CFA (red) injected animals. **(C)** Bar graphs of morphological parameters of SC neurons for saline (blue) and CFA (red) injected animals. **(D)** Bar graphs of electrophysiological parameters of SC neurons for saline (blue) and CFA (red) injected animals. Statistical significance was determined by two-tailed Student's t-tests. Error bars indicate SEM. * $p < 0.05$.

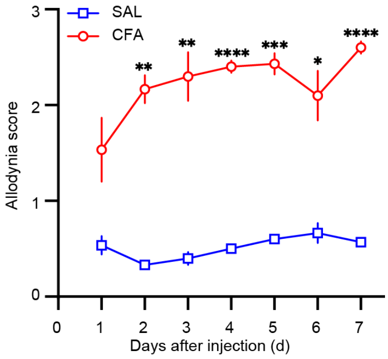


**Figure S9. Allodynia scores of noxious pinprick stimulation.** Pinprick responses noted for 6 trials, with a 5 min interval between each trial for saline (blue) and CFA (red) injected animals. The responses were scored as: 0 = paw lift; 1 = sustained lifting of the paw towards the body; 2 = strong lateral lifting above the level of the body; and 3 = flinching or licking of the affected paw (allodynia score) [20]. Statistical significance was determined by two-way ANOVA followed by Sidak's multiple comparison. Error bars indicate SEM. * $p < 0.05$, ** $p < 0.01$, *** $p < 0.001$, **** $p < 0.0001$.

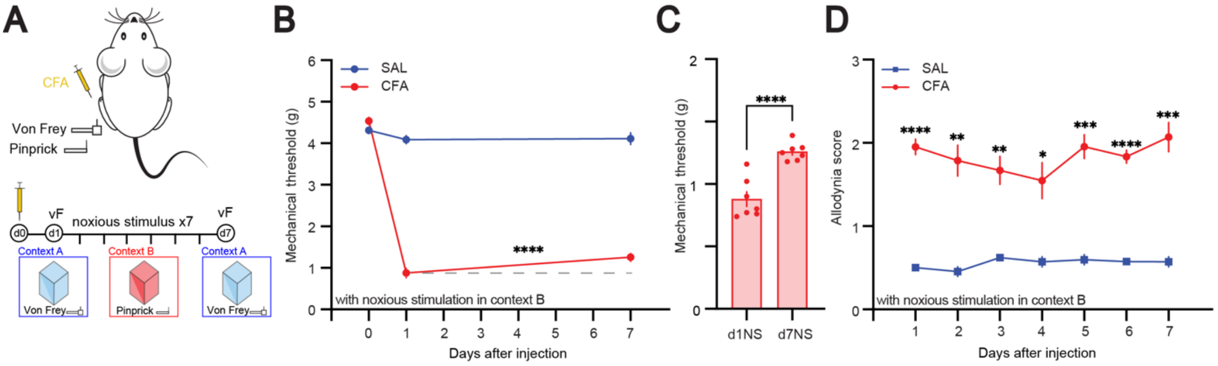


**Figure S10. Repeated noxious stimulation of the hind paws during peripheral inflammation has context-independent analgesic effects**. **(A)** Sketch and timeline of the CFA model of inflammatory pain. Von Frey testing was performed in context A, while daily noxious pinprick stimulation to both paws was conducted in a different context B, i.e. in a different room and visually distinct enclosure. **(B)** Mechanical threshold measured with an electronic von Frey device in context A over time for CFA (red) and saline (SAL, blue) injected animals subjected to daily noxious stimulation by pinprick in context B. Repeated noxious stimulation even in a different environment reduced the hypersensitivity in CFA injected mice on day 7. Dashed line indicates withdrawal threshold at day 1. **(C)** Bar graph of mechanical threshold of mice at d1 and d7 after CFA injection with daily noxious pinprick stimulation in context B for one week. **(D)** Allodynia scores of noxious pinprick stimulation in context B over time. Statistical significance was determined by two-tailed Student's t-tests in C and by two-way ANOVA followed by Sidak's multiple comparison in B and D. Error bars indicate SEM. * $p < 0.05$, ** $p < 0.01$, *** $p < 0.001$, **** $p < 0.0001$.

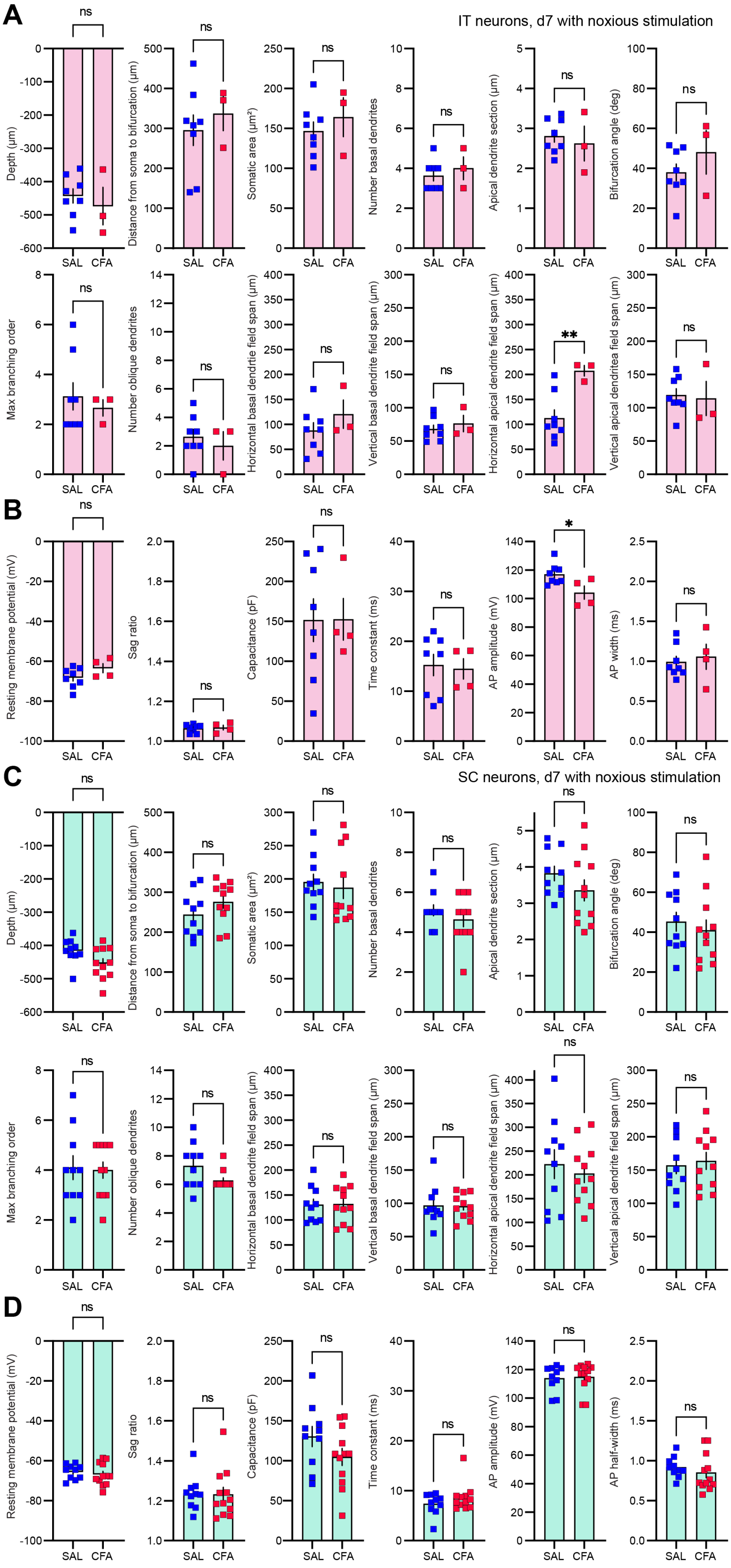


**Figure S11. Comparison of morphological and electrophysiological features of randomly patched PNs 7 days after saline or CFA injection and daily noxious stimulation that were post-hoc classified as IT and SC neurons. (A)** Bar graphs of morphological parameters of IT neurons for saline (blue) and CFA (red) injected animals. **(B)** Bar graphs of electrophysiological parameters of IT neurons for saline (blue) and CFA (red) injected animals. **(C)** Bar graphs of morphological parameters of SC neurons for saline (blue) and CFA (red) injected animals. **(D)** Bar graphs of electrophysiological parameters of SC neurons for saline (blue) and CFA (red) injected animals. Statistical significance was determined by two-tailed Student's t-tests. Error bars indicate SEM. * $p < 0.05$, ** $p < 0.01$.

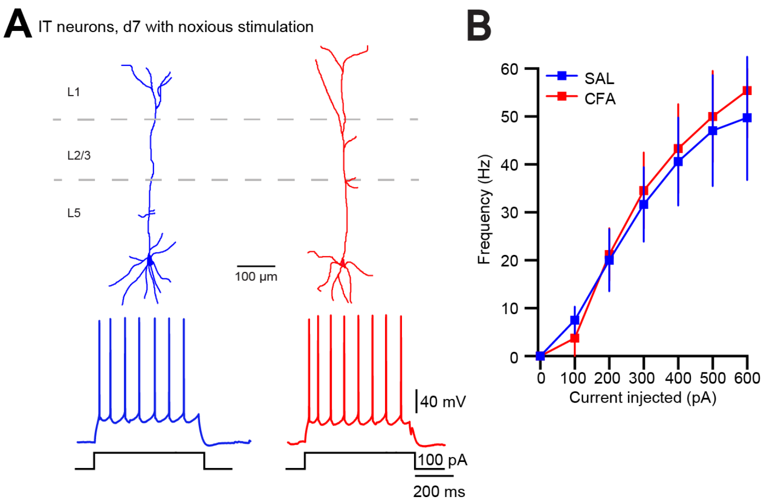


**Figure S12. Example of IT PNs recorded 7 days after saline or CFA injection and daily noxious stimulation. (A)** Reconstructions of representative IT neurons from saline and CFA injected mice on day 7 with daily noxious stimulation (above) and their corresponding firing pattern evoked by a 600 ms current injection of 100 pA (below). **(B)** Average F-I curves for the two conditions.

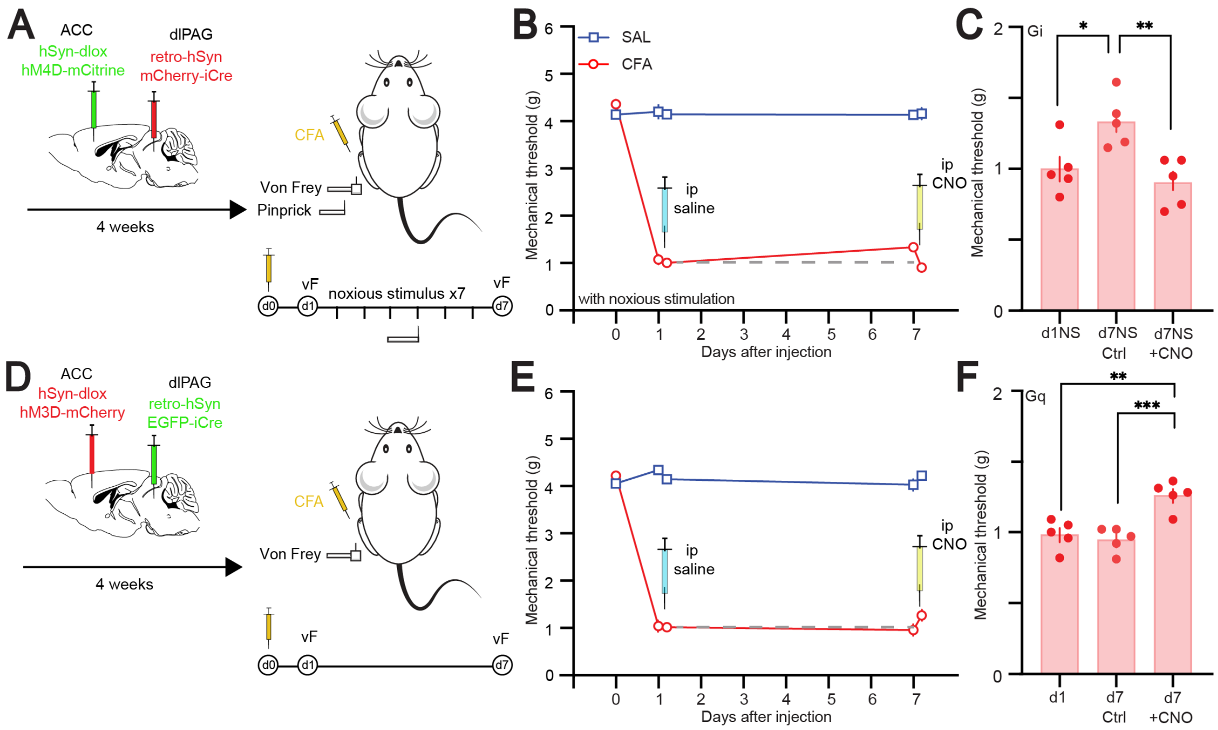


**Figure S13**. **ACC-to-dlPAG projecting SC neurons modulate pain behavior. (A)** Left, viral targeting strategy to express hM4D-mCitrine in ACC neurons projecting to dlPAG for chemogenetic silencing of this SC pathway. Right, sketch and timeline of the CFA model of inflammatory pain used for behavioral testing. Animals were subjected to daily noxious pinprick stimulation to both paws in this set of experiments. **(B)** Mechanical threshold measured with an electronic von Frey device over time for CFA (red) and saline (SAL, blue) injected animals subjected to daily noxious stimulation by pinprick. On day 7, mechanical sensitivity was assessed before and after i.p. injection of CNO to activate the inhibitory DREADD in the ACC-to-dlPAG pathway. Dashed line indicates withdrawal threshold at day 1. **(C)** Bar graph of mechanical threshold of mice at d1 and d7 after CFA injection subjected to daily pinprick stimulation and expressing the inhibitory DREADD before and after CNO injection. **(D)** Left, viral targeting strategy to express hM3D-mCherry in ACC neurons projecting to dlPAG for chemogenetic activation of this SC pathway. Right, sketch and timeline of the CFA model of inflammatory pain used for behavioral testing. Animals did not receive any intermediate interventions in this set of experiments. **(E)** Mechanical threshold measured with an electronic von Frey device over time for CFA (red) and saline (SAL, blue) injected animals subjected to no intermediate interventions. On day 7, mechanical sensitivity was assessed before and after i.p. injection of CNO to activate the excitatory DREADD in the ACC-to-dlPAG pathway. Dashed line indicates withdrawal threshold at day 1. **(F)** Bar graph of mechanical threshold of mice at d1 and d7 after CFA injection without intermediate interventions and expressing the excitatory DREADD before and after CNO injection. Statistical significance was determined by Two-way ANOVA followed by Sidak's multiple comparison. Error bars indicate SEM. * $p < 0.05$, ** $p < 0.01$, *** $p < 0.001$.

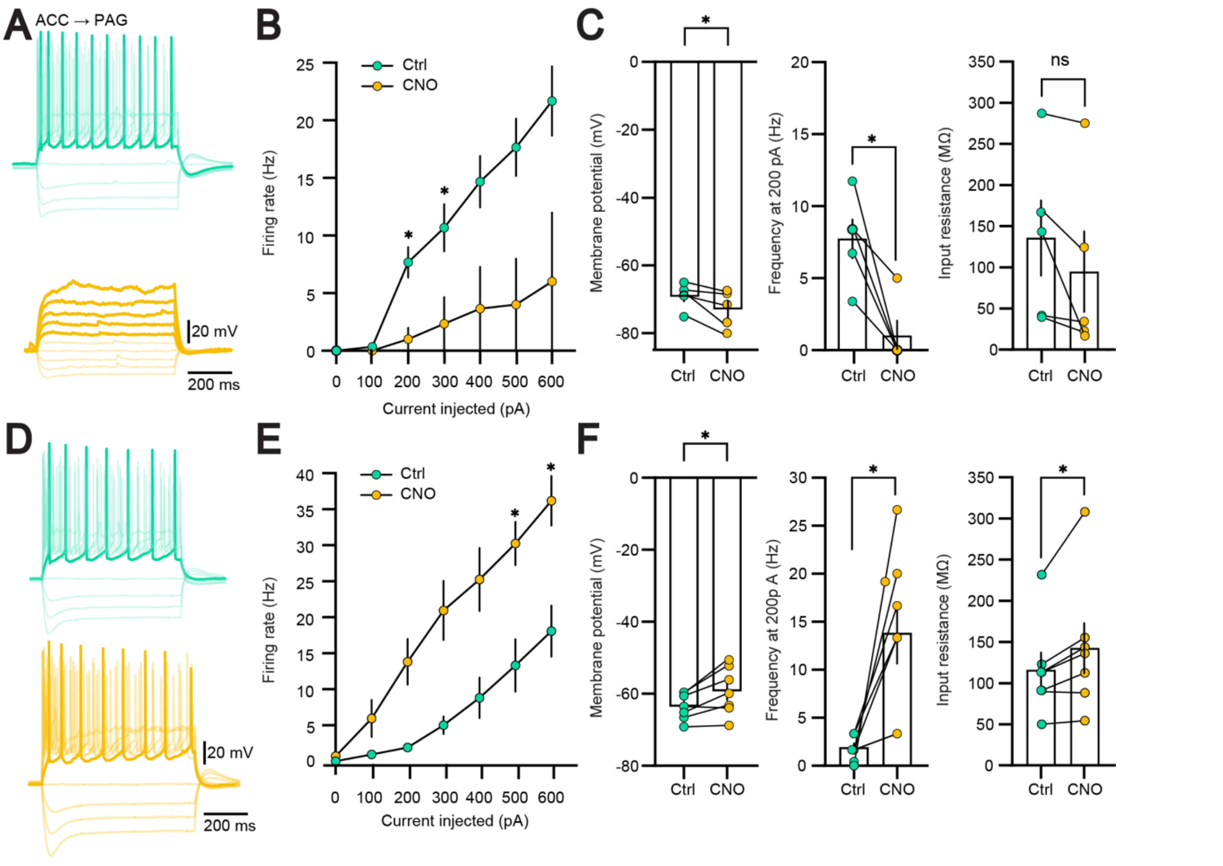


**Figure S14. Influence of Gi and Gq-DREADD activation on cellular excitability of L5 PNs projecting from ACC to vlPAG.** **(A)** Example traces of L5 PNs projecting to the vlPAG expressing the inhibitory DREADD hM4Di in response to current step injections before and 10 min after CNO (10 µM) application. **(B)** Corresponding F-I curves showing the firing rates for increasing current injections for before (green) and after CNO application (yellow). **(C)** Bar graphs of individual values of resting membrane potential, frequency of APs at 200 pA and input resistance. **(D)** Example traces of L5 PNs projecting to the vlPAG expressing the excitatory DREADD hM3Di in response to current step injections before and 10 min after CNO (10 µM) application. **(E)** Corresponding F-I curves showing the firing rates for increasing current injections for before (green) and after CNO application (yellow). **(F)** Bar graphs of individual values of resting membrane potential, frequency of APs at 200 pA and input resistance. Statistical significance was determined by paired two-tailed Student's t test for C, F and two-way ANOVA followed by Sidak's multiple comparison in B, E. Error bars indicate SEM. * $p < 0.05$, ** $p < 0.01$, *** $p < 0.001$, **** $p < 0.0001$.

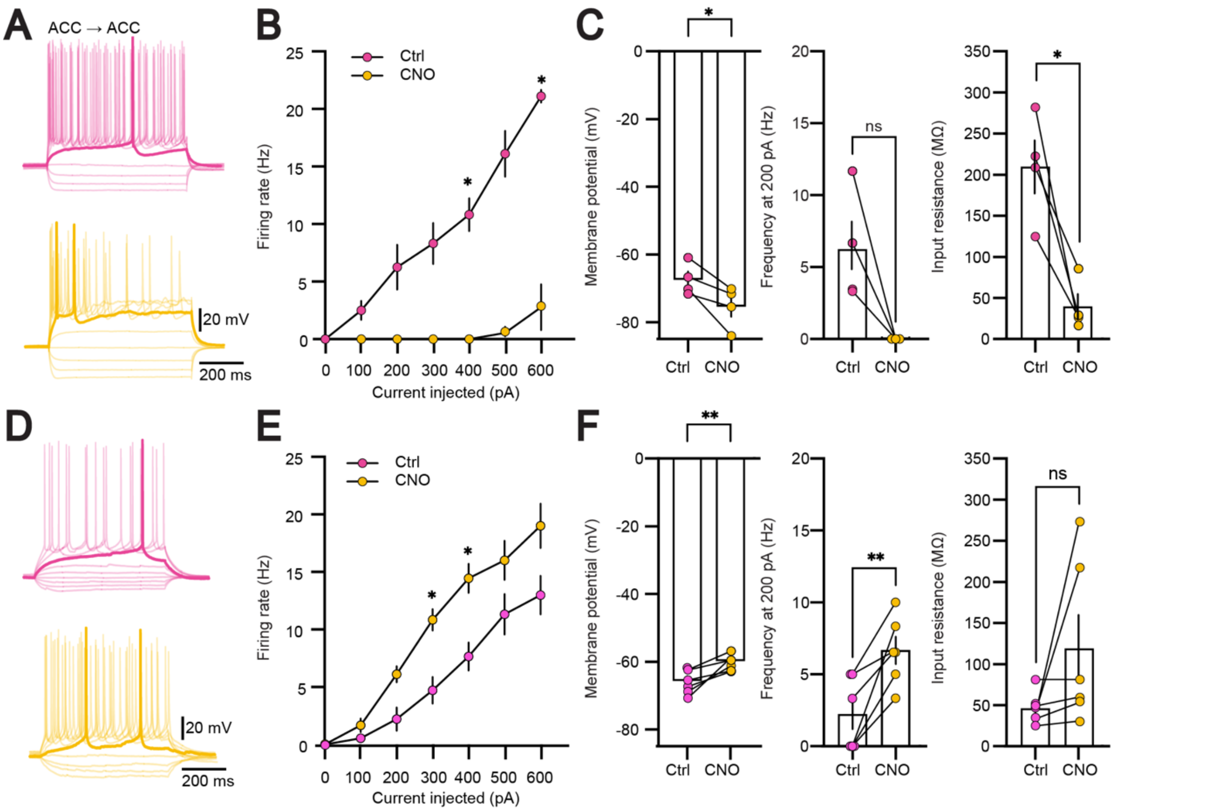


**Figure S15. Influence of Gi and Gq-DREADD activation on cellular excitability of L5 PNs projecting from iACC to cACC.** **(A)** Example traces of L5 PNs projecting to the cACC expressing the inhibitory DREADD hM4Di in response to current step injections before and 10 min after CNO (10 µM) application. **(B)** Corresponding F-I curves showing the firing rates for increasing current injections for before (magenta) and after CNO application (yellow). **(C)** Bar graphs of individual values of resting membrane potential, frequency of APs at 200 pA and input resistance. **(D)** Example traces of L5 PNs projecting to the vlPAG expressing the excitatory DREADD hM3Di in response to current step injections before and 10 min after CNO (10 µM) application. **(E)** Corresponding F-I curves showing the firing rates for increasing current injections for before (magenta) and after CNO application (yellow). **(F)** Bar graphs of individual values of resting membrane potential, frequency of APs at 200 pA and input resistance. Statistical significance was determined by paired two-tailed Student's t test for C, F and two-way ANOVA followed by Sidak's multiple comparison in B, E. Error bars indicate SEM. * $p < 0.05$, ** $p < 0.01$, *** $p < 0.001$, **** $p < 0.0001$.
